# Stiff‐Stilbene Ligands Target G‐Quadruplex DNA and Exhibit Selective Anticancer and Antiparasitic Activity†

**DOI:** 10.1002/chem.201905753

**Published:** 2020-04-28

**Authors:** Michael P. O'Hagan, Pablo Peñalver, Rosina S. L. Gibson, Juan C. Morales, M. Carmen Galan

**Affiliations:** ^1^ School of Chemistry University of Bristol Cantock's Close Bristol BS8 1TS UK; ^2^ Instituto de Parasitología y Biomedicina “López Neyra” Consejo Superior de Investigaciones Científicas (CSIC) PTS Granada, Avenida del Conocimiento 17 18016 Armilla, Granada Spain

**Keywords:** antiparasitic agents, antitumor agents, DNA, G-quadruplexes, molecular recognition

## Abstract

G‐quadruplex nucleic acid structures have long been studied as anticancer targets whilst their potential in antiparasitic therapy has only recently been recognized and barely explored. Herein, we report the synthesis, biophysical characterization, and in vitro screening of a series of stiff‐stilbene G4 binding ligands featuring different electronics, side‐chain chemistries, and molecular geometries. The ligands display selectivity for G4 DNA over duplex DNA and exhibit nanomolar toxicity against *Trypasanoma brucei* and HeLa cancer cells whilst remaining up to two orders of magnitude less toxic to non‐tumoral mammalian cell line MRC‐5. Our study demonstrates that stiff‐stilbenes show exciting potential as the basis of selective anticancer and antiparasitic therapies. To achieve the most efficient G4 recognition the scaffold must possess the optimal electronics, substitution pattern and correct molecular configuration.

## Graphical abstract

**Stiff scaffolds**: Stiff‐stilbene has very recently emerged as a promising scaffold for the design of selective G‐quadruplex DNA ligands. We demonstrate the importance of substituent chemistry and molecular geometry on the activity of this novel ligand series. Compounds exhibit up to 700‐fold selective toxicity to parasitic organisms and 30‐fold selective toxicity to cervical cancer cells over a non‐tumoral mammalian cell model.

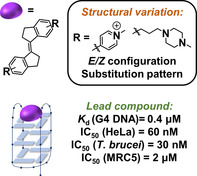

## Introduction

G‐quadruplexes (G4) are a class of nucleic acid secondary structure that form from sequences rich in guanine. In contrast to the classical duplex structure stabilized by Watson–Crick base pairing, the nucleic acid strand folds to create a stacked arrangement of G‐tetrads: square‐planar ensembles of four guanine residues stabilized by Hoogsteen hydrogen bonding and coordination to a central cation, such as Na^+^ or K^+^.^1^ These structures receive significant attention as potential therapeutic targets.^2^ Of particular interest is the quadruplex formed by the human telomeric sequence; telomerase expression is upregulated in cancer cells and partly responsible for cellular immortality by preventing telomere shortening, leading to uncontrolled proliferation.^2^, ^3^, ^4^ Furthermore, G4s are found in the promoter regions of several genes associated with the development of cancer (e.g., *c‐myc*,^5^ *BCL2* 6 and *c‐kit* 7), where stabilization of the folded G4 by ligands is proposed to inhibit the binding of transcription factors leading to downstream silencing of oncogene expression.^8^

More recently, we reported the identification of putative G4‐forming sequences in the genomes of the protazoan parasites *Trypanosoma brucei* and *Leishmania major*.^9^ Both organisms contain frequent occurrences of the human telomeric sequence^10^ in addition to several further unique G4s. For example, the EBR1 sequence occurs 33 times in the *T. brucei* genome and was subsequently demonstrated to form a stable quadruplex under physiological conditions in biophysical studies. The sequence occurs in genomic regions coding for several proteins including a cysteine peptidase and a purine transporter.^9^ G4s therefore present a potential opportunity as a target for novel antiparasitic therapies, for which there is an urgent need for further development.^11^ The *T. brucei* parasite, responsible for the Human African Trypanosomiasis (HAT) disease, endangers 69 million people across Sub‐Saharan Africa,^12^, ^13^ and existing therapies suffer from severe limitations including drastic side effects^14^ and emerging drug resistance^15^ in the parasitic strains. G4 ligands have long been studied as the basis of anticancer and antiviral therapeutics, but their potential as antiparasitic agents has been neglected until recently.^9^, ^16^ The handful of compounds explored to date are primarily naphthalene diimide derivatives, already widely studied as the basis of potential anticancer drugs. Though the activities are promising, the identification of further DNA‐binding chemotypes capable of exerting selective antiparasitic activity is of critical relevance to exploring this therapeutic hypothesis.

In a recent study, we identified a novel G4‐binding chemotype derived from stiff‐stilbene, **1**, the first example of a G4 ligand derived from this scaffold.^17^ Whilst ligand **1** induces high thermal stabilization of the potassium form of human telomeric DNA, the same compound causes the unfolding of the antiparallel form of the same sequence formed in sodium buffer. These intriguing activities suggested stiff‐stilbene as a promising scaffold for the design of potent and selective G4‐binding agents, prompting us to consider the potential applications of these derivatives as anticancer and antiparasitic agents. Towards this end, we synthesized further analogues of the previously reported compound and investigated the binding of these new derivatives using FRET thermal melting assay to a small library of G4 DNA sequences that are relevant in the targeting of cancers and parasitic infections. Binding was additionally validated through circular dichroism spectroscopy, UV/Vis titration studies and NMR spectroscopy. We then took initial steps to validate the therapeutic utility of these compounds by examining their toxicity and localization (intracellular uptake) in parasitic cultures and mammalian cells. Our results suggest a striking selectivity of the lead compounds for the target pathologies and recommend this class of DNA‐binding molecule as a candidate for further development of a new generation of anticancer and antiparasitic therapeutic leads.

## Results and Discussion

### Design and synthesis of stiff‐stilbene ligands

**Figure 1:**
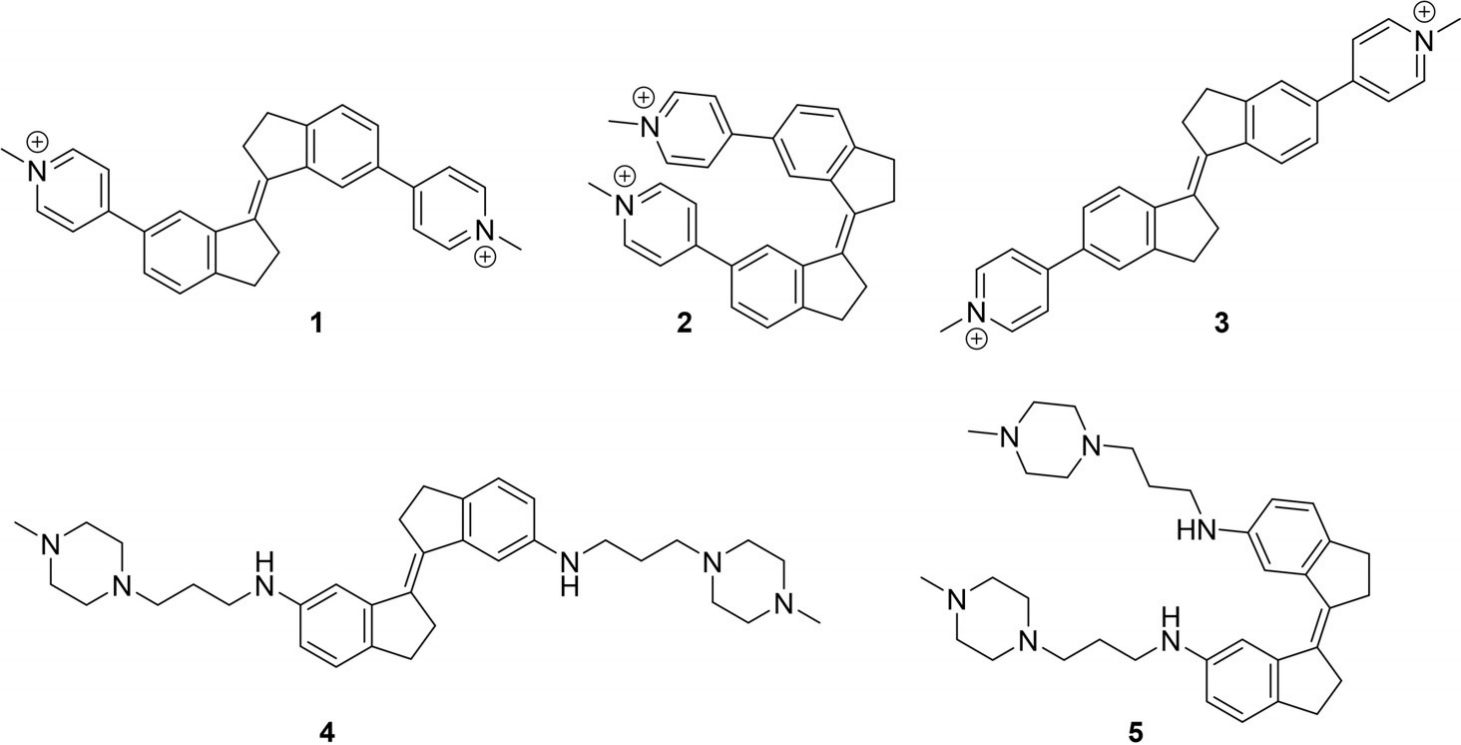
Structures of the stiff‐stilbene G4 ligands studied in this work.

**Scheme 1:**
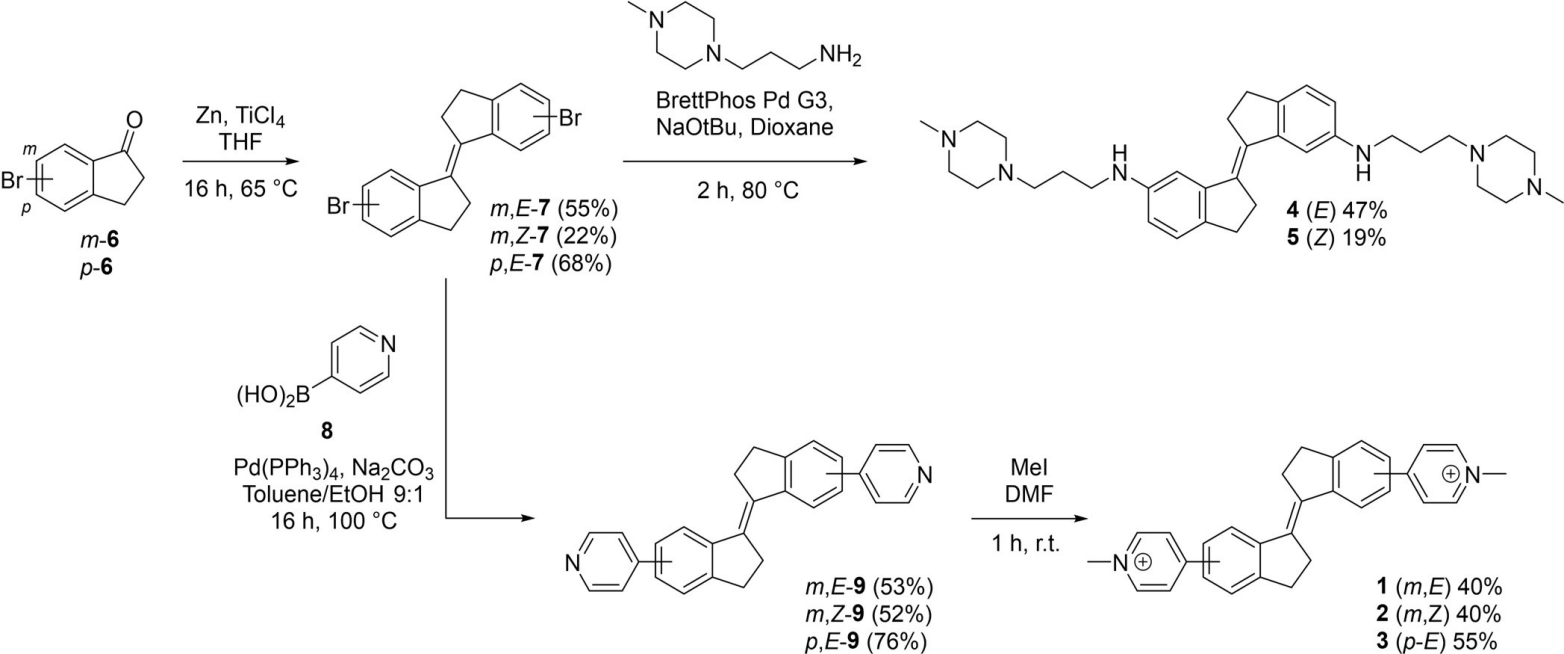
General synthetic route to stiff‐stilbene G4 ligands.

To evaluate the effect of ligand structure on the G4 binding properties and antiproliferative activities of stiff stilbene ligands, we prepared a small collection of compounds designed to investigate these effects (Figure 1). These derivatives incorporate a variety of stilbene stereochemical configuration, electronic effects, substitution pattern, and length and nature of the lateral groups. In particular, we were keen to examine the effect of exchanging the rigid methylpyridinium moiety in the previously reported compounds (**1** and **2**) for a more flexible amine‐derived side‐chain (compounds **4** and **5**), previously demonstrated by several groups to confer good G4 affinity by forming electrostatic interactions with the G4 grooves when protonated at physiological pH.^18^, ^19^ Meanwhile, the relocation of the pyridinium group to an alternative position on the scaffold (compound **3**) significantly alters the ligand shape from a more compact S‐shaped molecule to an extended linear structure, potentially influencing the ability to interact with G‐tetrads as well as modifying the electronic structure of the molecule. Finally, we were keen to further examine the effects of the configuration of the central stilbene core on G4 binding and in vitro activity by a more detailed comparison of the *E* and *Z* forms of the ligands (**1** vs. **2** and **4** vs. **5**). With routes to compounds **1** and **2** already established,^17^ related ligand **3** was synthesized using an analogous protocol (Scheme 1). Briefly, McMurry coupling of 5‐bromoindanone *p*‐**6** afforded bromide *p*‐**7**, which was coupled to 4‐pyridylboronic acid **8** using standard Suzuki conditions to afford intermediate *p*‐(*E*)‐**9**. Methylation proceeded smoothly to afford compound **3**, which was easily purified by trituration in acetone. Preparation of methylpiperazine analogues **4** and **5** proceeded straightforwardly by Buchwald–Hartwig amination of bromides *m*‐(*E*)‐**7** and *m*‐(*Z*)‐**7** with 1‐(3‐aminopropyl)‐4‐methylpiperazine, and the desired compounds **4** and **5** were obtained as the trifluoroacetate salts following purification by HPLC. Full synthetic procedures and characterization of the compounds is provided in the Supporting Information.

### Thermal melting assays

**Figure 2:**
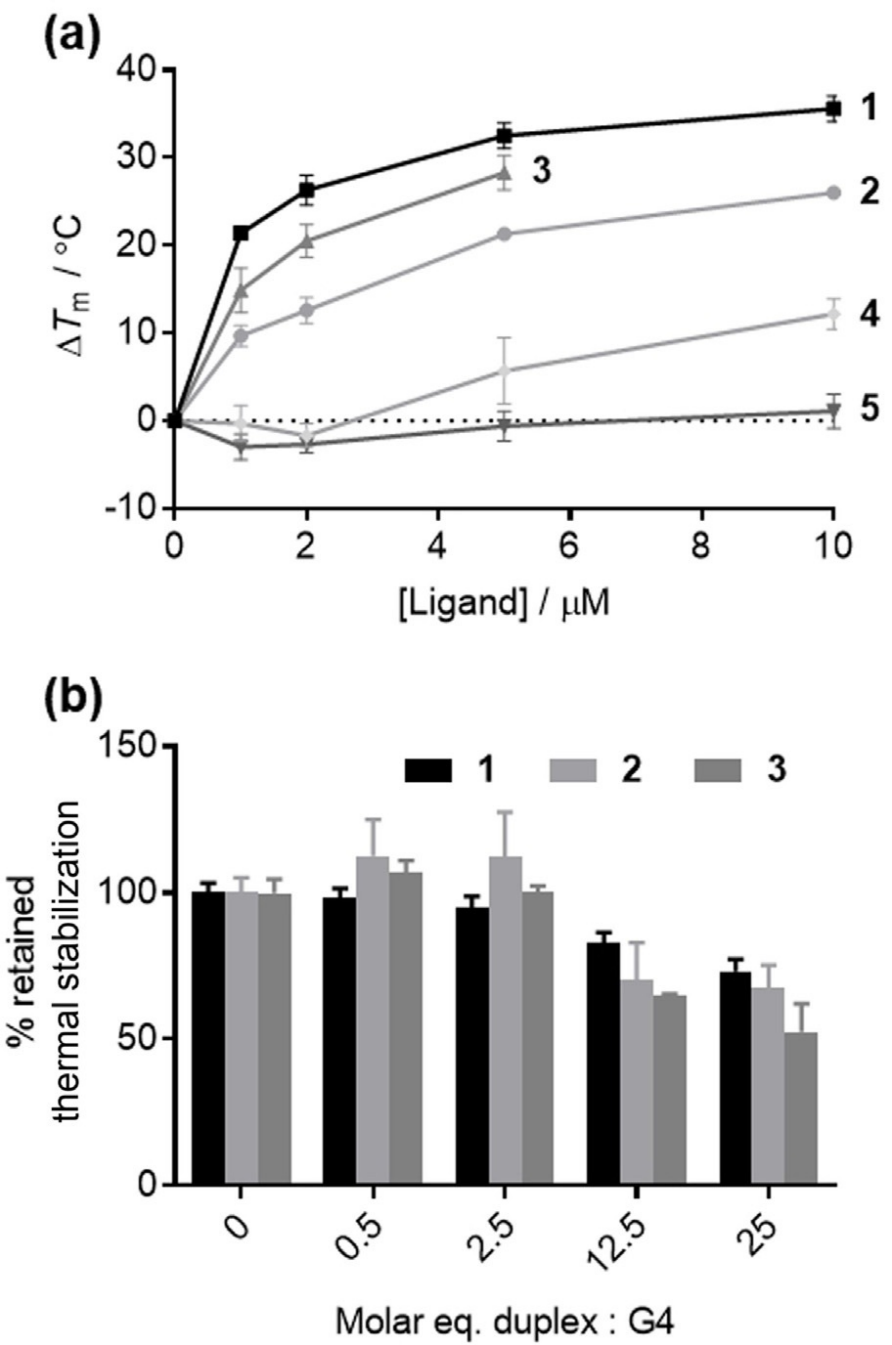
a) Dependence of Δ*T*_m_ of F21T (K^+^) on the concentration of each ligand. Owing to high ligand fluorescence, Δ*T*_m_ of ligand **3** could not be extracted above 5 μm. b) Retained thermal stabilization of F21T K^+^ by 1 μm ligands **1**–**3** in the presence of increasing concentrations of ds26, a competitor duplex DNA hairpin.

With the ligand series in hand, we initially sought to compare their ability to stabilize a variety of G‐quadruplex and duplex DNA structures with previously reported compounds by means of a fluorescence‐based thermal melting assay.^20^ Briefly, the DNA sequences of interest were conjugated to a fluorescence donor (FAM) and to an acceptor (TAMRA) at the 3′‐ and 5′‐ends, respectively. When folded into secondary structures, the proximity of the donor and acceptor results in quenching of the donor fluorescence by energy transfer (FRET) to the acceptor. Upon raising the temperature, the secondary structure denatures, causing the acceptor and donor to move apart, observed as an increase in donor fluorescence. The resulting curves (see the Supporting Information, Figures S1–S5) indicate the characteristic melting temperature (*T*_m_) of the sequence. Ligands that stabilize the folded structure cause the *T*_m_ to increase relative to that of the sequence without added ligand. The resulting difference in melting temperature (Δ*T*_m_) indicates the ability of the ligand to stabilize the folded structure (see the Supporting Information for full details of the experimental protocol). New ligands **3**–**5** were evaluated in this assay against the telomeric sequence F21T in potassium and sodium buffers and the FmycT sequence in potassium buffer. A duplex DNA hairpin (F10T) was also included to assess the ability of the ligands to discriminate between the different types of DNA secondary structure. Furthermore, all ligands **1**–**5** were screened against the newly available Febr1T G4 sequence found in the genome of *T. brucei*.^9^ The Δ*T*_m_ values for all ligands (at 5 μm concentration) are displayed in Table 1, and dependence of Δ*T*_m_ on ligand concentration is displayed in Figure 2 a for the potassium form of F21T, the sequence for which the ligands appear to be most affine. Concentration‐dependence curves for the other DNA sequences are provided in the Supporting Information (Figures S6–S9).

**Table 1:** Thermal stabilization (Δ*T*_m_) induced in G4 and duplex DNA by 5 μm ligands **1**–**5**.^[a]^

Ligand	Δ*T*_m_ [°C]				
	F21T K^+^	F21T Na^+^	FmycT	Febr1T	F10T
**1** ^[b]^	33	26	29	22	5
**2** ^[b]^	21	13	18	9	3
**3**	28	19	30	17	3
**4**	6	0	5	4	0
**5**	0	0	0	2	0

[a] F21T=5′‐FAM‐GGGTTAGGGTTAGGGTTAGGG‐TAMRA‐3′; FmycT=5′‐FAM‐TTGAGGGTGGGTAGGGTGGGTAA‐TAMRA‐3′; Febr1T=5′‐FAM‐GGGCAGGGGGTGATGGGGAGGAGCCAGGG‐TAMRA‐3′; F10T=5′‐FAM‐TATAGCTATA‐HEG‐TATAGCTATA‐TAMRA‐3.′ The DNA concentration was 0.2 μm. Representative melting curves and error bars are shown in the Supporting Information (Figures S1–S9) [b] Δ*T*_m_ values for ligands **1** and **2** against F21T, FmycT and F10T were reported previously.^17^

From our initial screen, the most striking feature is that the pyridinium ligand class (**1**–**3**) show superior binding when compared to the flexible methylpiperazine‐derived ligands (**4**–**5**). For example, ligand **4** only displays significant stabilization in the quadruplex sequences at 5 μm ligand concentration and above, whilst all three pyridinium derivatives **1**–**3** remain potent down to 1 μm ligand concentration (Figure 2 a and the Supporting Information, Figure S6–S8). Given that many potent G4 ligands, such as the well‐explored naphthalene diimide (NDI) family, feature flexible basic side‐chains of the type exemplified by **4** and **5**,^18^, ^19^, ^21^ it is somewhat surprising that such a design feature does not appear to confer high G4 stabilizing ability to the stiff‐stilbene scaffold, despite this core displaying a clear ability to serve as the basis of selective G4 ligands (as exemplified by compounds **1**–**3**). Compounds in the NDI series functionalized with alkyl chains bearing methyl piperazine termini have been found to bind G4 by forming stacking interactions between the NDI core and external tetrads of the G4, with the basic side chains residing in the G4 grooves.^19^ The comparatively poor performance of the stiff‐stilbene analogues of these compounds suggests this scaffold is comparatively ineffective at achieving such a binding mode and perhaps the high stabilization displayed by ligands **1**–**3** may originate from alternative binding modes, such as groove binding rather than end‐stacking (see further discussion below).

Both *Z* ligands (**2** and **5**) are comparatively weak G4 binders in comparison to their *E* counterparts. Whilst the *E* ligand **4** induces appreciable stabilization in the potassium form of F21T K^+^ (Δ*T*_m_ at 5 μm=6 °C), *Z* isomer **5** induces a negligible stabilization at the same concentration. A similar effect was previously observed for ligands **1** and **2**. Given the significantly different molecular geometrical configurations of the *Z* and *E* stiff stilbene core, these results suggest that the olefin configuration of the central scaffold is critical in determining the activity in this class of compound, and is independent of the nature of the lateral groups. Meanwhile, the Δ*T*_m_ values observed for pyridinium ligand **3** are, in general, significantly lower than those for compound **1**. This suggests that the more compact arrangement of **1** proves superior for G4 binding when compared with the extended linear configuration of ligand **3**.

It is important to highlight that all ligands **1**–**5** induce only negligible to minor stabilization on the duplex DNA model F10T at concentrations that induce significant stabilization of G4. This indicates the stiff‐stilbene scaffold possesses an inherent selectivity for binding to G4 over duplex sequences. Given the particularly high activity of compounds **1**–**3**, we were keen to verify the observed selectivity of these compounds by running a competition experiment in which the effect of increasing concentrations of unlabelled duplex DNA (ds26) on the Δ*T*_m_ of the G4 sequences is measured. Under such conditions, off‐target binding is observed as reduction in the Δ*T*_m_ value relative to that obtained in the absence of the competing species. All three pyridinium ligands **1**–**3** discriminate most effectively between the F21T K^+^ sequence and duplex DNA (Figure 2 b). Even at 25 molar equivalents of ds26, over 50 % of the thermal stabilization induced by ligands **1**–**3** is retained for this sequence. The ligands appear to be significantly less selective against the same sequence in sodium‐containing buffer (see the Supporting Information, Figure S10), particularly ligand **2**, for which the thermal stabilization induced by the ligand is lost entirely at 12.5 molar equivalents of ds26 competitor and above. This behavior is reflective of the lower affinity of the ligands for F21T in sodium‐containing buffer observed in Table 1. Both ligands **1** and **3** (the more potent G4 ligands) retain over 50 % of the induced thermal stabilization of the FmycT sequence (see the Supporting Information, Figure S11) at 25 molar equivalents of ds26, confirming a high degree of selectivity for these ligands. On the other hand, ligand **2** is again less selective, and a gradual erosion of the induced stabilization on increasing the concentration of duplex competitor is observed. Against the FebrT found in the *T. brucei* genome, ligand **1** outperforms ligands **2** and **3** for selective targeting of the G4 structure over duplex DNA (see the Supporting Information, Figure S12). Taken together, the results of Table 1 and the competition assays across the panel of G4s indicate compound **1** as the lead ligand in the series in terms of both the magnitude of the induced thermal stabilization of G4, and its general selectivity for G4 DNA in favor of the double‐stranded secondary structure.

### Circular dichroism spectroscopy and UV/Vis titration studies

To further examine the nature of interaction of ligands **1**–**5** with G4 topologies, we employed a combination of circular dichroism (CD) spectroscopy and UV/Vis titration studies (techniques commonly used for the study of G4/ligand interactions).^22^ Both approaches have the advantage that modification of the oligonucleotide sequence with artificial fluorophores is not necessary, allowing validation of binding effects against natural sequences where the folding topology is well‐validated by structural studies. These studies therefore allow inference of possible binding modes along with quantification of binding affinity under physiologically‐relevant conditions. Prior to undertaking these studies, we first validated that all ligands were stable to photoisomerization/photodegradation under the assay conditions (see the Supporting Information, Figure S13). In this study, we chose to examine the binding of the ligands to the hybrid (telo23, potassium) form of the telomeric sequence,^23^ since this sequence occurs in both human and parasitic genomes and is the most relevant topology of this G4 in vivo owing to the high concentration of potassium inside cells.^24^

**Figure 3:**
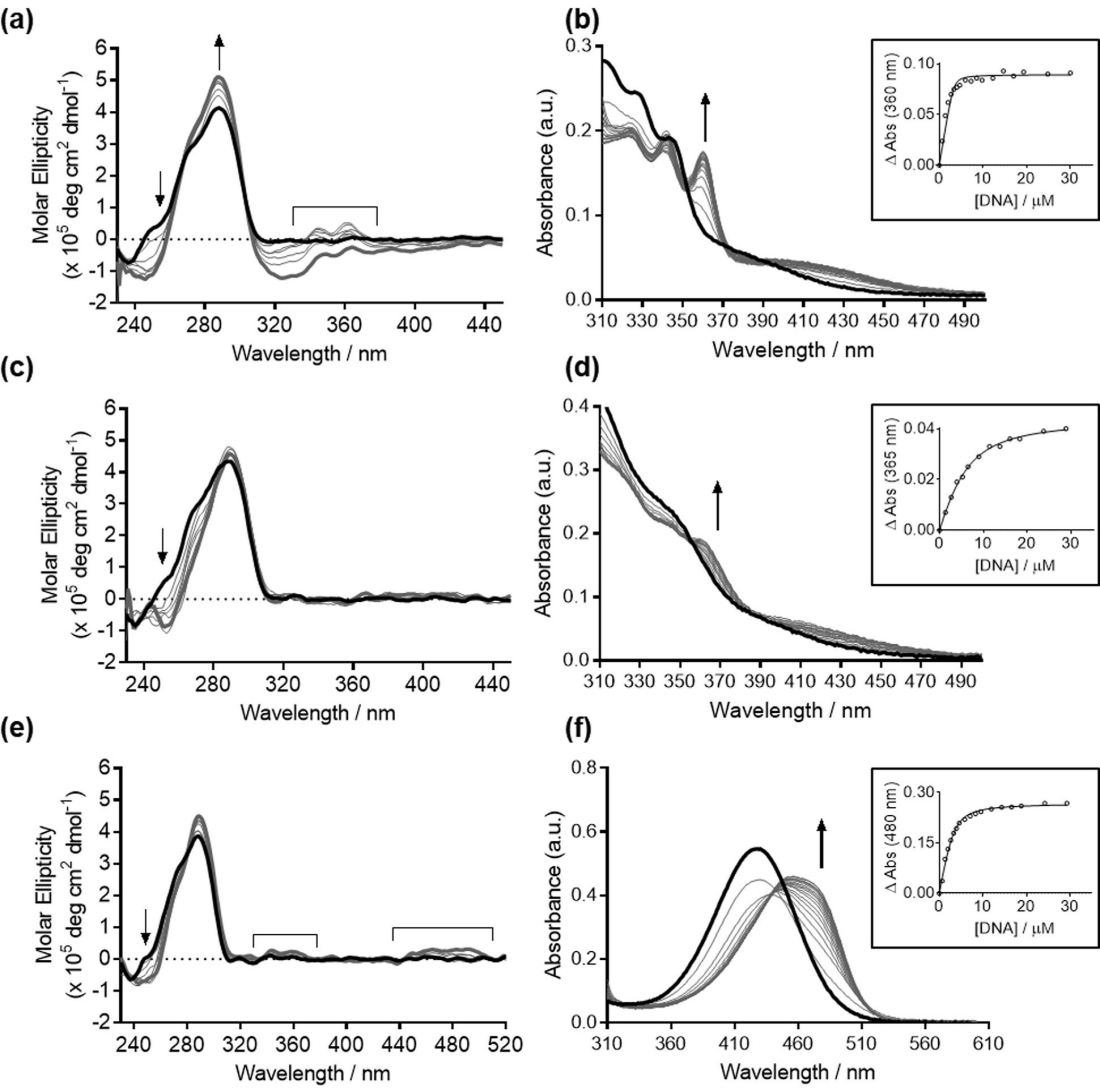
Circular dichroism and UV/Vis titrations of ligands **1**, **2**, **3**. CD spectra of telo23 (4.2 μm, black traces) titrated with 0 (black trace) to 7 equiv (dark gray trace) of the ligands a) **1**, c) **2**, and e) **3**. Intermediate titration points are shown in light gray. Induced CD signals in the ligand regions are marked with a square bracket. UV/Vis spectra of ligands b) **1**, d) **2**, and f) **3** (10 μm) titrated with telo23. The insets show fitting of the data to independent and equivalent sites binding models to yield the dissociation constants. Data for compound **1** were reported previously.^17^

The circular dichroism spectrum of telo23 is characterized by a positive band at 290 nm, and a weaker shoulder band at 260 nm indicative of a predominant hybrid G4 topology.^25^ Binding of the pyridinium derivatives **1**–**3** is evidenced by hyperchromicity in the positive band at 290 nm (Figure 3 a, c and e) and the increased intensity of the negative band at 240 nm. The effect is most striking for compound **1** (as previously reported^17^), which is consistent with this compound being the more potent of the three pyridinium ligands investigated in the current study. These changes indicate an overall stabilization of the native G4 fold by compound **1**. However, whilst the 260 nm shoulder band is preserved upon titration with ligand **1** (Figure 3 a), this band disappears upon titration with ligand **2** (Figure 3 c), suggesting a shift in folding equilibrium in favor of an alternative G4 topology. Whilst it is not possible to draw detailed conclusions from CD data alone, subsequent NMR studies (see below) indicate that the equilibrium mixture of major and minor species formed by telo23 under the experimental conditions shifts to favor a single species on addition of ligand **2**. The CD spectral features present in the telo23/ligand **2** complex are superficially representative of an antiparallel‐folded G4, but more detailed studies are necessary to truly establish the precise structure of the telo23/**2** complex. Only very weak induced CD signals are observed in the ligand region for compound **3**, in contrast to the more evident induced CD signals induced by ligand **1**, suggesting that whilst ligand **1** binds through groove binding modes,^17^, ^26^ end stacking modes are available for ligand **3**. Lesser (though still significant) spectral perturbations were observed for ligand **4** (see the Supporting Information, Figure S14a) indicating the weaker affinity of this ligand for G4. No perturbation of the CD spectra of telo23 was observed for ligand **5** (see the Supporting Information, Figure S14b), corroborating the results from the FRET assay, where no stabilization of G4 was observed over the range of concentrations studied.

Quantification of the binding affinity of ligands **1**–**5** to hybrid G4 was performed using UV/Vis titration studies (Table 2). The resulting titrations and representative isotherms are shown in Figures 3 b, d and f, and in the Supporting Information (Figures S15–S19), in which the absorbance spectrum of the ligand was measured upon titration with telo23 G4. Apparent binding isotherms and stoichiometries were determined by fitting the observed hyperchromic shifts to an independent‐and‐equivalent‐sites binding model (see the Supporting Information).^27^, ^28^ Strikingly, the observed affinities mirror the trends observed in the thermal melting assays, with compound **1** again emerging as the most potent G4 ligand. Ligand **5**, which displayed negligible effect on the stability of G4, induced only subtle perturbations to the UV/Vis spectrum of telo23 (see the Supporting Information, Figure S19), indicating weak interaction and meaning the dissociation constant could not be reliably determined. These results confirm that the critical nature of the stilbene configuration in G4 recognition, with *E* compounds **1** and **3** exhibiting micromolar G4 affinity, whilst *Z* ligand **2** displays affinity two orders of magnitude lower. Notably, a striking bathochromic shift (ca. 30 nm) is observed in the spectrum of ligand **3** (Figure 3 f). This effect is indicative that an end‐stacking ligand binding mode is present, where the energy of the π–π* transition responsible for the Soret band is lowered by the interaction of the ligand chromophore with the G‐tetrad.^29^ Such marked shifts are not observed for related ligand **1**, for which the CD data is indicative of groove binding modes.^17^

**Table 2:** Apparent ligand/telo23 dissociation constants from UV/Vis‐titration experiments.^[a]^

Ligand	*K*_d_ [μm]	Ligand/G4^[b]^
**1**	0.4	3
**2**	70	2
**3**	2	3
**4**	70	1
**5**	n.d.^[c]^	n.d.^[c]^

[a] For full details and data, see the Supporting Information. [b] Number of ligand binding sites assumed in binding model. [c] *K*_d_ too weak for accurate determination.

**Figure 4:**
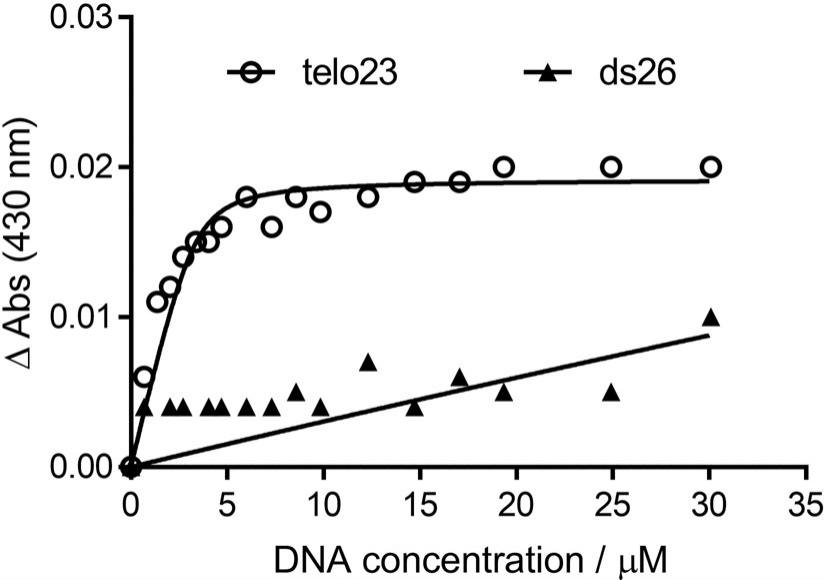
The *λ*=430 nm binding isotherms for ligand **1** titrated with telo23 G4 and ds26 duplex DNA sequences indicating the availability of a specific binding mode to G4 that is not available for duplex DNA.

To further investigate the G4/duplex selectivity observed for lead compound **1**, we undertook a further titration study of this compound with duplex DNA (ds26) to examine the origin of the selectivity between the two DNA species observed in the FRET assay (Figure S20). Changes in the UV‐region of the ligand **1** absorbance spectrum also occur upon titration with ds26, albeit to a lesser degree than with the G4 species. We propose these perturbations arise from electrostatic interactions between the cationic ligand and the negatively charged DNA‐phosphate backbone, which are likely to be relatively independent of DNA secondary structure when compared to specific steric binding modes. More strikingly, the induction of hyperchromicity in the shoulder band in the visible region of the ligand spectrum (centered on 430 nm) by telo23, is barely observed upon titration with duplex DNA (Figure 4). Therefore, it appears that the telo23 G4 provides a further ligand binding mode that is not accessible in the duplex sequence. We propose that this structure‐specific binding mode is responsible for the overall G4 selectivity observed in the melting assays.

### NMR studies

**Figure 5:**
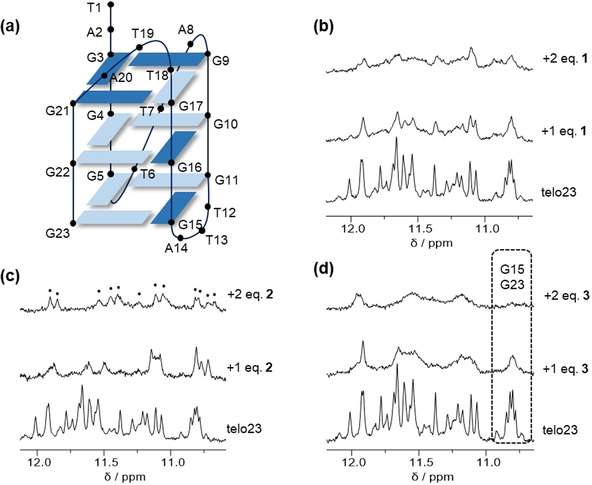
NMR titration studies of telo23 in potassium‐containing buffer with ligands **1**–**3**. a) Representation of the major G4 species formed under the experimental conditions. NMR spectra of telo23 in the absence of ligand and with increasing equivalents of ligand b) **1**, c) **2**, and d) **3**. In c) the 12 G‐tetrad imino resonances (indicating shift to a single conformation) are marked. In d) the disappearance of G15/G23 signals (lower G‐tetrad) is indicated.

To provide initial structural insights into the data obtained in the UV/Vis and circular dichroism titration experiments, and provide preliminary validation of the proposed binding modes of ligands **1**–**3** to telo23 G4, we undertook 1D ^1^H imino NMR experiments (Figure 5). Spectra were interpreted using assignments of the resonances of the major hybrid‐fold species previously reported by Patel et al.^23^ Significant line broadening of the imino resonances can be observed upon titration with ligand **1 (**Figure 5 b). Such broadening effects could be attributed to the strong binding of this ligand, resulting in intermediate‐to‐slow exchange between free and bound ligand states on the NMR timescale. All imino signals broaden to a similar degree and remain distinguishable, suggesting interactions with specific G‐tetrad residues do not dominate in the association of ligand **1** with G4 and providing additional evidence for the groove binding mode inferred from the CD titrations. Ligand **3** (Figure 5 d) also induces spectral line broadening, though signals associated with the lower G‐tetrad (G15/G23) disappear entirely, suggesting stacking (or possibly intercalative) interactions with this part of the G4 are important in the binding of ligand **3**. Meanwhile, line broadening is much less significant for ligand **2**. This ligand exhibits weaker binding to the G4 (see above) and therefore faster exchange between bound and unbound ligand states on the NMR timescale can be expected. This allows significant chemical shift perturbations to be observed in the imino resonances of the G4 upon addition of the ligand, indicating that this ligand may also interact with the G4 target through association with the G‐tetrads. Interestingly, while the unbound G4 sequence exists as a mixture of major and minor conformations, complexation with ligand **3** appears to favor a single conformation, with only 12 distinct imino signals visible at 2:1 ligand:G4 stoichiometry (indicated in Figure 5 c) versus the more complex spectrum for the G4 sequence in the absence of the ligand, which represents a mixture of major and minor folded species, as previously reported.^23^ This structural perturbation may explain the disappearance of the shoulder band in the CD spectrum of telo23 upon titration with ligand **3**. In conjunction with the data obtained in the CD and UV/Vis titrations, we infer that ligand **1** does interact primarily through groove binding, whereas stacking interactions are more important in the binding of ligands **2** and **3**.

### Toxicity studies

Having demonstrated a range of binding affinities for stiff‐stilbene ligands **1**–**5** to G4 DNA, we were keen to make an initial assessment of the performance of these compounds as therapeutic agents. In particular, we were interested to examine whether the in vitro biological activity of the compounds correlated with their DNA binding properties. We measured the viability of parasitic and mammalian cell cultures in the presence of increasing doses of compounds **1**–**5**. Activity was first measured against both *T. brucei* and *L. major,* with the MRC‐5 line chosen as a non‐tumoral cell model for comparison. To our delight, compounds **1**–**5** exhibited potent toxicity to *T. brucei* with GI_50_ values in the nanomolar range for all compounds (Table 3 and the Supporting Information, Figure S21). Critically, the compounds are significantly less toxic against the non‐tumoral MRC‐5 cells with selectivity index of up to 700‐fold, suggesting a promising therapeutic window for these compounds as anti‐trypasanomal agents. However, the fact that ligand **5** also exhibits high toxicity despite its poor affinity for G4 suggests the mechanism of toxicity is unlikely to be related to G4 recognition in the case of this organism. Further studies are underway to investigate the origin of the high selectivity index of these compounds for *T. brucei*, which are significantly higher than those observed in related studies.^9^, ^16^ Though appearing to exert a lower toxicity to the *L. major* parasite (Table 3 and Supporting Information, Figure S22), ligands **1** and **3** (which demonstrated the strongest affinity for G4), show sub‐micromolar efficacy against this organism. The weaker G4 ligands (compounds **2**, **4** and **5**) were significantly less efficacious, suggesting a potential role for G4 recognition in their mechanism of action in this case. The selectivity index for *L. major* is significantly lower than observed in the case of *T. brucei*, but the values are comparable to those observed for other G4 ligands screened against this organism.^9^

**Table 3:** Viability assay data for ligands **1**–**5** against parasitic (*T. brucei* and *L. major*) cultures and non‐tumoral (MRC‐5) mammalian cells after 72 h incubation^[a]^

Ligand	GI_50_ [μm]			Selectivity index GI_50_ (MRC‐5)/GI_50_ (parasite)	
	MRC‐5	*T. brucei*	*L. major*	*T. brucei*	*L. major*
**1**	3.1±1	0.029±0.0005	0.64±0.1	110	4.8
**2**	12±1	0.043±0.0001	11±2	280	1.1
**3**	0.35±0.1	0.0061±0.002	0.11±0.03	60	3.2
**4**	10±1	0.036±0.001	16±0.4	280	0.6
**5**	9.0±1	0.012±0.005	2.3±0.07	750	3.9

[a] Measured by Alamar blue assay (MRC‐5, *T. brucei*) or MTT assay (*L. major*). See the Supporting Information for full details and dose‐response curves

We next examined the potential of the compounds to serve as anticancer agents. At three‐day exposure, all compounds exhibited low toxicity (GI_50_=10–100 μm) to HeLa cervical cancer cells (see the Supporting Information, Table S1 and Figure S23a). However, previous work by others has identified that a significantly longer exposure time to G4‐binding compounds is often necessary to observe toxic effects, since a mechanism of action involving telomere shortening theoretically requires several population doublings to take effect.^30^ We therefore subjected both HeLa and MRC‐5 cells to a longer‐term (seven day) exposure of compounds **1**–**5** (Table 4 and the Supporting Information, Figures S23b and S24b). Strikingly, whereas the toxicity towards MRC‐5 was comparable with that observed at shorter‐term exposure (GI_50_=10–100 μm), the cancerous cells became significantly more susceptible to lower doses of compound **1** (GI_50_=62 nm), which, as we demonstrated above, is the most potent G4 ligand in our compound series. Indeed, this increase in potency returns a selectivity index of 29 at long‐term exposure. We rationalize that the fact that the toxicity to MRC‐5 cells does not significantly depend on exposure time for **1** could be attributed to the telomerase negative nature of this cell line, resulting in less susceptibility to the effects of telomeric G4 binding ligands. This ligand therefore shows significant promise as the basis of a potential G4‐mediated selective cancer therapeutic and is worthy of future extensive screening and mechanism of action studies. The toxicity of the weaker G4 ligands **2**, **4** and **5** are comparable (within one order of magnitude) to the values observed at short‐term exposure. This indicates that although these compounds do elicit some cytotoxicity to mammalian cells, a long‐term mechanism of action, as observed for ligand **1**, is not present. This is perhaps explained by the relatively low G4 affinity of these compounds observed in the titration studies and thermal melting assays. Compound **3** also displayed significantly stronger toxicity to HeLa cells at long‐term exposure, but its acute toxicity to MRC‐5 cells (retained on long‐term exposure) yields only a very modest selectivity index of 4. We propose that the poor discrimination between the cancerous and non‐cancerous cell lines might result from binding to duplex DNA, since the FRET assays determined this compound to be less selective for G4‐DNA than ligand **1**.

**Table 4:** Viability assay data for ligands **1**–**5** against tumoral (HeLa) and non‐tumoral (MRC‐5) mammalian cells after 7 day incubation.^[a]^

Ligand	GI_50_ [μm]		Selectivity index
	MRC‐5 (7 d)	HeLa (7 d)	GI_50_ (MRC‐5)/ GI_50_ (HeLa)
**1**	1.8±0.5	0.062±0.01	29
**2**	20±5	5.2±2	3.8
**3**	0.46±0.1	0.11±0.03	4.2
**4**	18±0.5	16±0.3	1.1
**5**	3.2±0.07	3.1±2	1.0

[a] Measured by Alamar blue assay. See the Supporting Information for full details and dose‐response curves.

### Confocal microscopy

**Figure 6:**
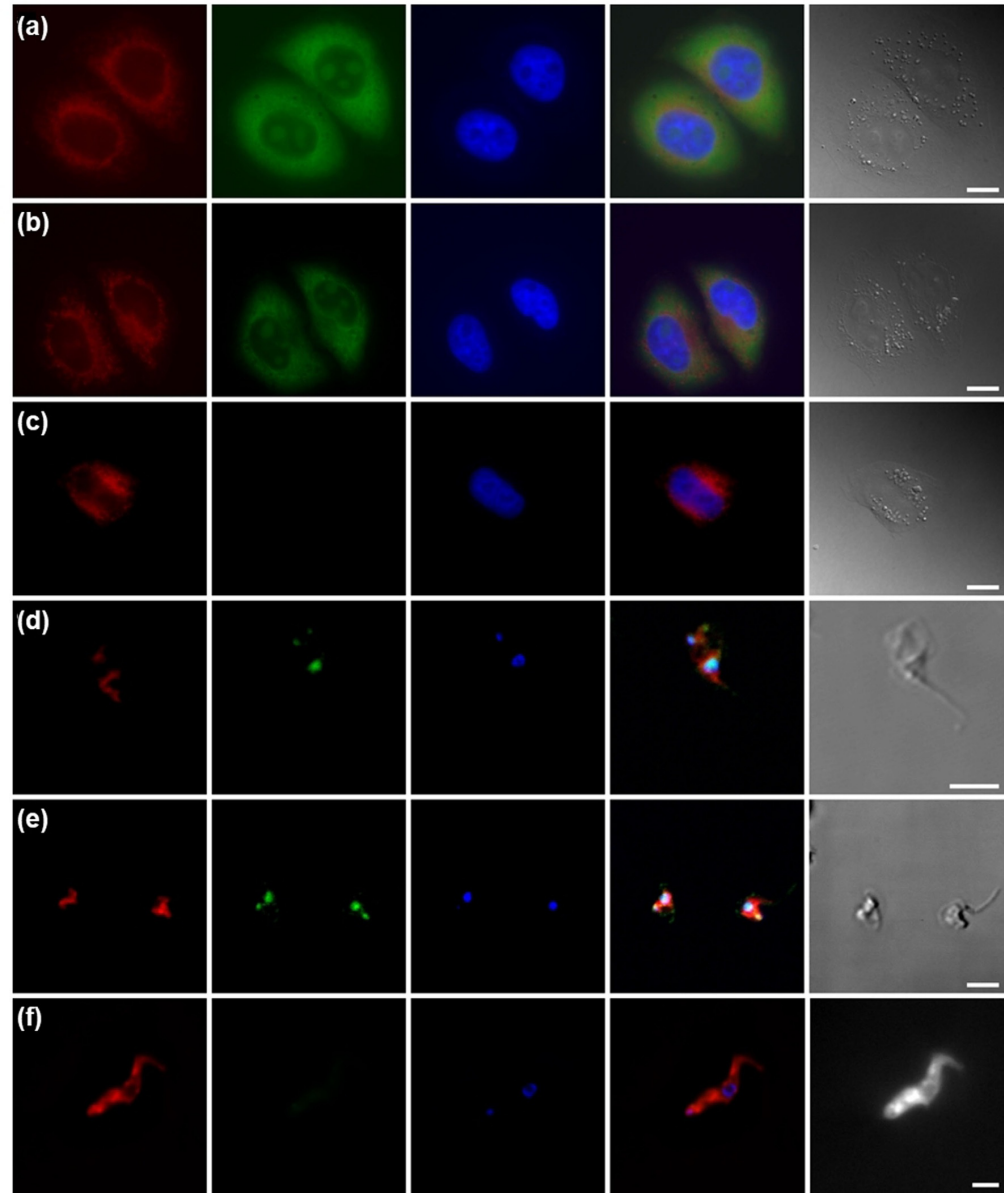
Fluorescence microscopy images of HeLa cells and *T. brucei* parasites after incubation with (respectively) 0.5 or 1 μm ligand **3** for a, d) 30 or b, e) 120 min. c) Control cells and f) parasites are also displayed. Visualization panes from left to right: mitochondria (Mitotracker Red), ligand **3**, nucleus (DAPI), co‐localization image, bright‐field image. Scale bar: 20 μm (cells) and 5 μm (parasites).

As a step towards validating the mechanism of action of our compounds and more concretely establish the intracellular localization of the ligands within the cell lines studied, we examined the uptake of these compounds by mammalian cells and parasites through microscopy studies. Unfortunately, lead compound **1** was not sufficiently fluorescent to visualize using this technique. We therefore undertook localization studies on compound **3**, which has photophysical properties much better suited to visualization in cells (λ_em_=550 nm, Supporting Information, Figure S25). After 30 min incubation at 37 °C, significant uptake of compound **3** in both *T. brucei* and HeLa cells was observed. The ligand was mainly localized in the nucleolus and the cytoplasm of HeLa cells, and partially in the mitochondria (Figure 6 a). In *T. brucei*, ligand **3** was mainly found in the nucleus and the kinetoplast (Figures 6 d). Similar locations patters were observed at longer (2 h) incubation times (Figure 6 b and e), and in *L. major* and MRC‐5 cells (see the Supporting Information, Figure S26). These results suggest that pyridinium stiff‐stilbene G4‐ligands can reach DNA harboring sites and therefore possibly target G4 structures.

## Conclusions

G4 nucleic acids continue to offer exciting potential as a target against a range of disease pathologies. However, no G4‐targeting drug has reached the clinic to date. The identification and development of new ligand scaffolds that display promising bioactivities is in high demand and will form the basis of new G4‐based drug discovery projects. We have examined a selection of the structural features that govern the binding of a novel series of stiff‐stilbene ligands to nucleic acid targets. We show that ligand **1** appears to have the optimal molecular configuration, electronics and substitution pattern for efficient G4 recognition amongst this first generation of compounds. Moreover, we have demonstrated that **1** has a high level of selectivity for G4 structures, and anticipate that this selectivity could be further improved through the development of subsequent generations of G4‐binding molecules based on this emergent G4‐binding chemotype. Furthermore, we have shown for the first time that stiff‐stilbene derivatives demonstrate high toxicity both to parasitic organisms and cancerous cell lines, whilst they remain up to two orders of magnitude less toxic to a non‐tumoral model. Critically, in the case of the cellular models, in vitro cytotoxicity to cancerous cells strongly correlates with G4‐binding activity, and dose response times indicate long‐term mechanisms of action that may include G4‐mediated pathways, such as telomerase inhibition. We believe this proof‐of‐concept study reveals intriguing activities that render stiff‐stilbene compounds an exciting lead scaffold for DNA‐targeted drug development. Towards this end, further investigations into the therapeutic mechanism of stiff‐stilbene ligands is warranted in order to interrogate the true biological targets and obtain more conclusive evidence regarding the potential G4‐mediated mode of activity. Efforts towards these goals are under active pursuit in our laboratory and progress will be reported in due course.

## Conflict of interest

The authors declare no conflict of interest.

## Supporting information

As a service to our authors and readers, this journal provides supporting information supplied by the authors. Such materials are peer reviewed and may be re‐organized for online delivery, but are not copy‐edited or typeset. Technical support issues arising from supporting information (other than missing files) should be addressed to the authors.

Supplementary
