# Supplementary material for: Stiff‐Stilbene Ligands Target G‐Quadruplex DNA and Exhibit Selective Anticancer and Antiparasitic Activity
Source: Chemistry. 2020 Apr 28;26(28):6224–33. doi: 10.1002/chem.201905753 (PMC7318697; doi:10.1002/chem.201905753)
Supplement: Supplementary file 1 — Supplementary [file CHEM-26-6224-s001.pdf]

Chemistry–A European Journal

Supporting Information

Stiff-Stilbene Ligands Target G-Quadruplex DNA and Exhibit Selective Anticancer and Antiparasitic Activity**

Michael P. O'Hagan,[a] Pablo Peñalver,[b] Rosina S. L. Gibson,[a] Juan C. Morales,*[b] and M. Carmen Galan*[a]

Contents

1 Experimental details ... 2
1.1 FRET melting assays ... 2
1.2 Circular dichroism titrations ... 3
1.3 Determination of apparent dissociation constants by UV-visible spectroscopy ... 4
1.4 NMR experiments ... 4
1.5 Cell culture ... 5
1.6 Cytotoxicity studies ... 5
1.7 Parasitic toxicity studies ... 6
1.8 Fluorescence microscopy ... 6
1.9 Confocal microscopy ... 7
2 Supplementary figures ... 8
3 Supplementary tables ... 20
4 Synthetic procedures and compound characterisation ... 20
5 NMR spectra of tested compounds ... 29
6 NMR spectra of novel synthetic intermediates ... 34
7 References ... 36

1 Experimental details

1.1 FRET melting assays

Fluorescence resonance energy transfer (FRET) melting assays were performed according to the procedure reported by De Cian and co-workers[1] using a Roche LightCycler 480 qPCR instrument. In these assays, the oligonucleotides of interest were obtained labelled at the 5′ and 3′ ends with FAM (a fluorescence donor) and TAMRA (a fluorescence quencher), respectively. In the folded state, proximity of the donor and quencher result in no observed fluorescence from FAM, since energy is transferred non-radiatively to TAMRA by FRET. As the temperature is raised and the secondary structure denatures, the fluorophores move further apart and hence the fluorescence signal increases. From the resulting curve, the characteristic melting temperature ($T_{1/2}$) is defined as that at which the normalised fluorescence signal equals 0.5. The change in melting temperature (ΔT_m) induced by a small molecule ligand compared to that of the oligonucleotide in the absence of ligand provides an indication of the ligand's ability to stabilise the G4 structure.

All oligonucleotides used were purchased from Eurogentec (Belgium), purified by HPLC and delivered dry. Oligonucleotide concentrations were determined by using the Beer-Lambert law from the UV-absorbance at 260 nm (measured using a NanoDrop 2000 Spectrophotometer from Thermo Scientific) and the oligonucleotide molar extinction coefficient at this wavelength (ε_{260}, calculated by the supplier). The oligonucleotides used were as follows:

DNA model	Sequence / extinction coefficient
F21T (human telomeric G4)	5′-FAM-GGGTTAGGGTTAGGGTTAGGG-TAMRA-3′
	ε_{260} = 268300
FmycT (c-myc promoter G4)	5′-FAM-TTGAGGGTGGGTAGGGTGGGTAA-TAMRA-3′
	ε_{260} = 290100
Febr1T (*T.brucei* G4)	5′-FAM-GGGCAGGGGGTGATGGGGAGGAGCCAGGG-TAMRA-3′
	ε_{260} = 349000
F10T (duplex)	5′-FAM-TATAGCTATA-HEG-TATAGCTATA-TAMRA-3′
	ε_{260} = 263600
ds26 (unlabelled duplex)	5′-CAATCGGATCGAATTCGATCCGATTG-3′
	ε_{260} = 253200

FAM = 6-carboxyfluorescein;

TAMRA = 6-carboxy-tetramethylrhodamine;

HEG = [(-CH_2CH_2O-$)_6$]

All sequences were annealed before use by heating for 2 minutes at 90°C and then placed immediately into ice. The final concentration of oligonucleotide was 200 nM in all cases. The buffer used depended on the sequence in question, for F21T in Na^+ conditions, the final buffer contained 100mm NaCl, and 10 mm Li cacodylate. For FmycT (K^+ conditions), 1 mM KCl, 99 mM LiCl and 10 mM Li cacodylate were used. For F21T in K^+ conditions, Febr1T and F10T, 10 mM KCl, 90 mM LiCl and 10 mM Li cacodylate were used. Ligand concentrations were either 1 µM, 2 µM, 5 µM or 10 µM. Each sample was tested in duplicate on the same plate, and each plate was repeated in at least duplicate to assess the reproducibility of all results. Oligonucleotide denaturation was detected by monitoring FAM fluorescence (λ_{ex} = 494 nm, λ_{em} = 518 nm). Appropriate control experiments were also carried out for each sample set. Data processing was carried out using Origin 9, with $\Delta T_{1/2}$ used to represent ΔT_m.

1.2 Circular dichroism titrations

Circular Dichroism (CD) titrations were recorded using a Jasco J-810 spectrometer fitted with a Peltier temperature controller. Measurements were taken in a quartz cuvette with a path length of 5 mm, at 20 °C, at a 100 nm / min scanning speed at 1 nm intervals, with a 1 nm bandwidth. The CD spectra were recorded between 800 and 200 nm, and baseline corrected for the buffer used. The oligonucleotide sequence used was telo23: (hybrid model, 5'-TAGGGTTAGGGTTAGGGTTAGGG-3').[2] The oligonucleotide was purchased from Eurogentec (Belgium), purified by HPLC and delivered dry. Oligonucleotide concentrations were determined as discussed in Section 1.1 using the appropriate molar extinction coefficients: ε_{260} (telo23) = 236500. The oligonucleotide was annealed before use by heating for 2 minutes at 90°C and then placed immediately into ice. The oligonucleotide was at a concentration of 4.2 µM which gave an OD of 1 and the buffer used was potassium phosphate (100 mM, pH 7.4). The ligand was added by aliquot from a 1mM stock solution in the appropriate buffer (containing 10% DMSO to ensure solubility). The reported spectrum for each sample represents the average of 3 scans. Data processing was carried out using Prism 7 with an 8-point second order smoothing polynomial applied to all spectra. Observed ellipticities were converted to mean residue ellipticity (θ) = deg cm^2 $dmol^{-1}$ (molar ellipticity).

1.3 Determination of apparent dissociation constants by UV-visible spectroscopy

Apparent dissociation constants for ligands **1**-**5** were determined through UV-visible spectroscopy titration experiments. UV spectra were recorded on a Thermo Scientific BIOMATE 3S UV-vis Visible Spectrophotometer at ambient temperature. Measurements were taken in a 3 mL quartz cuvette with a path length of 10 mm. The UV-visible spectra were recorded between 800 nm and 200 nm and baseline corrected for the buffer used. The concentration of ligand was fixed at 10 µM in a constant volume of 1.5 mL buffer. The buffer was potassium (telo23) phosphate (100 mM, pH 7.4). During the titration, aliquots of sample were removed and replaced with aliquots of oligonucleotide to give the required titration points (from a 100 µM stock solution in appropriate buffer containing also 10 µM ligand to maintain constant ligand concentration). NB: the oligonucleotide solution was annealed by heating to 90 °C for 2 minutes and then cooling on ice **prior** to the addition of ligand (to avoid annealing in the presence of ligand). Following addition, the solution was mixed thoroughly and the UV-visible spectrum was acquired immediately. Data were fitted to an independent-and-equivalent-sites binding model (Equation 1) using Prism 7 software, a full derivation of which is provided by (amongst others) Thordarson,[3] adapted to an independent and equivalent sites model by (amongst others) Buurma and Gade.[4] The stoichiometry of the complex (*N*) was chosen as the lowest integer value that provided a satisfactory fit.

Equation 1:

$$\Delta A = \varepsilon_{\Delta complex}([\text{complex}])$$

$$where:$$

$$[\text{complex}] = \frac{1 + N \cdot K_\text{a} \cdot [\text{DNA}]_{tot} + K_\text{a} \cdot [\text{ligand}]_{tot} - \sqrt{(1 + N \cdot K_\text{a} \cdot [\text{DNA}]_{tot} + K_\text{a} \cdot [\text{ligand}]_{tot})^2 - 4 \cdot N \cdot {K_\text{a}}^2 \cdot [\text{DNA}]_{tot} \cdot [\text{ligand}]_{tot}}}{2 \cdot K_\text{a}}$$

$\Delta A = absorbance\ change\ at\ each\ titration\ point\ relative\ to\ free\ ligand\ (observed\ parameter)$

$\varepsilon_{\Delta complex} =\ change\ in\ the\ molar\ extinction\ coefficient\ between\ free\ ligand\ and\ DNA/ligand\ complex\ (fitted\ parameter)$

$N = the\ binding\ stoichiometery\ of\ ligand\ to\ DNA\ (selected\ parameter)$

$K_\text{a} = the\ apparent\ association\ constant$ (fitted parameter), $K_\text{d} = \frac{1}{K_\text{a}}$

$[\text{DNA}]_{tot} = the\ concentration\ of\ added\ DNA\ (known\ parameter)$

$[\text{ligand}]_{tot} = the\ total\ ligand\ concentration\ (fixed\ parameter)$

1.4 NMR experiments

^{1}H NMR spectra of telo23 were recorded at 298 K using a 600 MHz Varian VNMRS spectrometer equipped with a triple resonance cryogenically cooled probe head. Samples of oligonucleotide were dissolved in 90%

H2O/10% D_2O containing 20 mM potassium phosphate (pH = 7.0) and 70mM potassium chloride. All experiments employed sculpted excitation water suppression. The final NMR samples contained 600 µL of 185 µM oligonucleotide. Samples were annealed before use by heating for 2 minutes at 90°C and then placed immediately into ice.

Aliquots of ligand (10 mM in DMSO-d_6) were added to the appropriate yield titration points, the sample was mixed thoroughly and NMR spectra were recorded immediately after the addition of ligand. Data were processed using MestReNova software (version 11.0.2). Resonances were assigned from data provided in the literature by Patel *et al.*[2]

1.5 Cell culture

MRC-5 cells (human lung fibroblasts) were grown in monolayer (37°C, 5% CO2 and 100% of humidity) in DMEM medium (1 g/L glucose), supplemented with 10% heat-inactivated Fetal Bovine Serum, 2 mM L-glutamine, 100 U/mL penicillin and 100 mg/mL streptomycin. HeLa cells were maintained at 37 °C and 5 % CO_2 in high glucose DMEM (4.5 g/L) supplemented with 10% hiFBS, 100 U/ml penicillin, 100 mg/ml streptomycin, 2 mM L-glutamine and non-essential amino acids (1X). Cells were cultured according to ATCC recommendations and were used for the experiments while in the exponential growth phase.

T. brucei (bloodstream forms, “single marker” S427 (S16)) were cultured at 37 °C, 5 % CO_2 in HMI-9 medium supplemented with 10% heat-inactivated fetal bovine serum (hiFBS, Invitrogen). *L. major* promastigotes (MHOM/IL/80/Friedlin) were cultured at 28 °C, 5 % CO_2 in modified RPMI-1640 medium (Invitrogen, Carlsbad, CA) with 10% hiFBS. Parasites were split every other day and maintained in their experimental growth phase (below 2 million parasites per mL)

1.6 Cytotoxicity studies

Cytotoxicity was measured through the alamarBlue assay (ThermoFisher scientific).[5,6] Briefly, 5x10^3 MRC-5 or HeLa cells were seeded in 96-wells plates (100uL/well) in the presence of increasing concentrations of ligands. After 72 hrs of incubation at 37 °C, 20 µL of Alamar Blue solution (110 ng/ml) were added to each well and cells were reincubated for 4 hrs at 37 °C. Then, 50 µL of 3% SDS were added to each well. The plate was incubated at 37 °C for an extra hour and then analysed by fluorescence at the Infinite F200 plate reader (TECAN Austria, GmbH). Excitation wavelength was fixed to 550nm and emission wavelength to 590nm. The

results are expressed as the concentration of compound that reduces cell growth by 50% versus untreated control cells (GI_{50}) using Prism software to fit the data to a sigmoidal curve. Data are presented as the average of two independent measurements all conducted in duplicate conditions.

1.7 Parasitic toxicity studies

The trypanocidal activity of the compounds was also assessed by the alamarBlue assay.[5,6] Briefly, $1x10^3$ BSF *T. brucei* were incubated in 96-wells plates alone or in the presence of increasing concentrations of compounds for 72 hrs (5% CO_2, 37°C) and processed described above. Data are presented as the average of three independent measurements all conducted in triplicate conditions.

The leishmaniacidal activity of the compounds on promastigotes *L. major* (MHOM/IL/80/Friedlin) was carried out as previously described.[7] Briefly, 4×10^6/mL promastigotes were incubated for 72 h at 28 °C in 96-well plates (50 µL/well) in the presence of increasing concentration of compounds. Cell proliferation was determined using a MTT-based assay (Sigma-Aldrich). Thus, after the 72 h incubation, 10 µL of MTT (5 mg/ml) were added to each well and parasites were reincubated for 4 hrs at 28°C. Then, 50 µL of 20% SDS were added to each well. The plate was then incubated at 37 °C for 4-16 hours and analysed at the Infinite F200 plate reader (TECAN Austria, GmbH). The absorbance was measured at a wavelength of 540 nm and the GI_{50} was calculated as described above. Data are presented as the average of two independent measurements all conducted in duplicate conditions.

1.8 Fluorescence microscopy

HeLa cells (20.000/ mL) and *T. brucei* parasites (10.000.000/ mL) were incubated with 0.5 and 1 µM ligand **3**, respectively in 0.5 mL of each respective medium (without FBS) for 30 and 120 min at 37 °C and 100% of humidity. Deep red mitotracker (400 nM) was also added to the incubation well for the last 30 minutes. Then the parasites were fixed with cold paraformaldehyde 4% for 30 min, washed twice in cold phosphate buffered saline (PBS), and processed by microscope observation. Cells were washed 5 times with room temperature PBS and fixed with paraformaldehyde 2% for 20 minutes. Two extra PBS washings and an ethanol immersion of the cover slide were also necessary prior to sample processing for microscopy. Prolong DAPI (3-4 µL) was used as mounting medium in both cases.

Images were acquired using a widefield Olympus ix81 microscope. Excitation was performed with the 350-450, 492-518 and 572-623 nm filters for DAPI, ligand **3** and Deep Red Mitotracker, respectively. The emission of ligand **3** was detected between 510 and 550 nm with a narrow filter, whereas a triple filter (437-474, 508-550 and 595-670 nm) was used to detect the fluorescence emission of both, DAPI and Mitotracker.

The images were deconvoluted using Huygens Professional image processing software from Scientific Volume Imaging (http://www.svi.nl). The merge of the images were made with Fiji software (https://fiji.sc/).

1.9 Confocal microscopy

MRC5 cells (20.000/ mL) and *Leishmania* parasites (10.000.000/ mL) were incubated with 5 and 1µM ligand **3**, respectively in 0.5 mL medium (without FBS) for 30 and 150 min at 100% of humidity and 37 or 28 °C, respectively. Deep red mitotracker (400 nM) was also added to the incubation well for the last 30 minutes. Then the cells and parasites were fixed and processed as for fluorescence microscopy studies.

Images were acquired using a Leica SP5 confocal microscope. Excitation was performed with the UV diode 405, 488 and 633 nm lasers for DAPI, ligand **3** and Deep Red Mitotracker, respectively. A photomultiplier was used to detect the emission of ligand **3** between 540 and 650 nm, whereas DAPI and Mitotracker were detected at 415-487 and 661-732 nm, respectively.

The images were deconvoluted using Huygens Professional image processing software from Scientific Volume Imaging (http://www.svi.nl). The merge of the images were made with Fiji software (https://fiji.sc/).

2 Supplementary figures

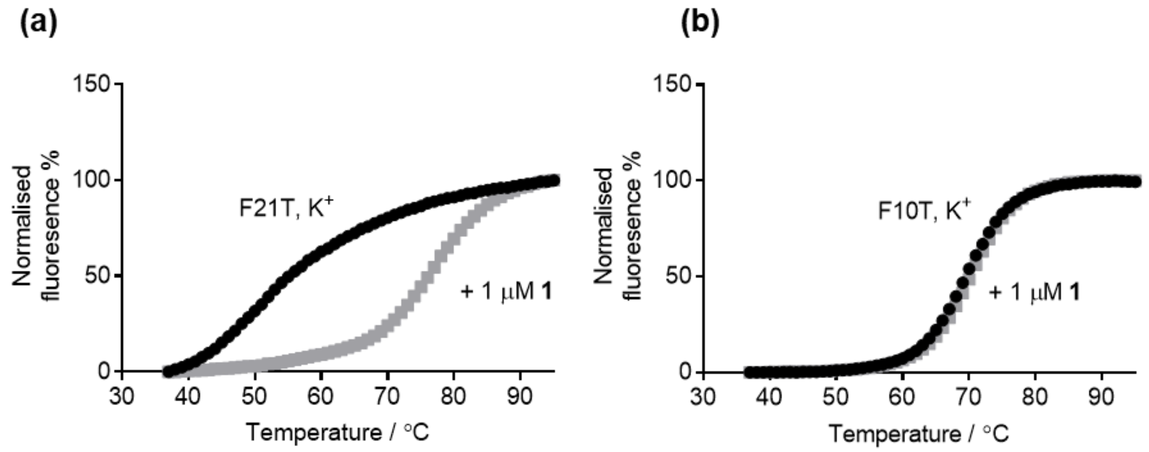

Figure S1: Representative FRET melting curves for ligand **1** against (a) F21T (K^+ form) and (b) F10T.[8]

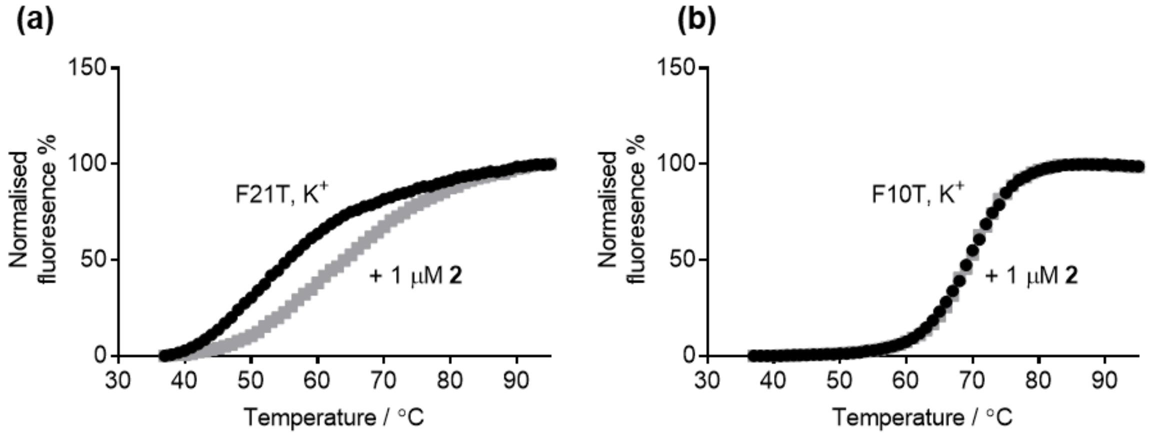

Figure S2: Representative FRET melting curves for ligand **2** against (a) F21T (K^+ form) and (b) F10T.

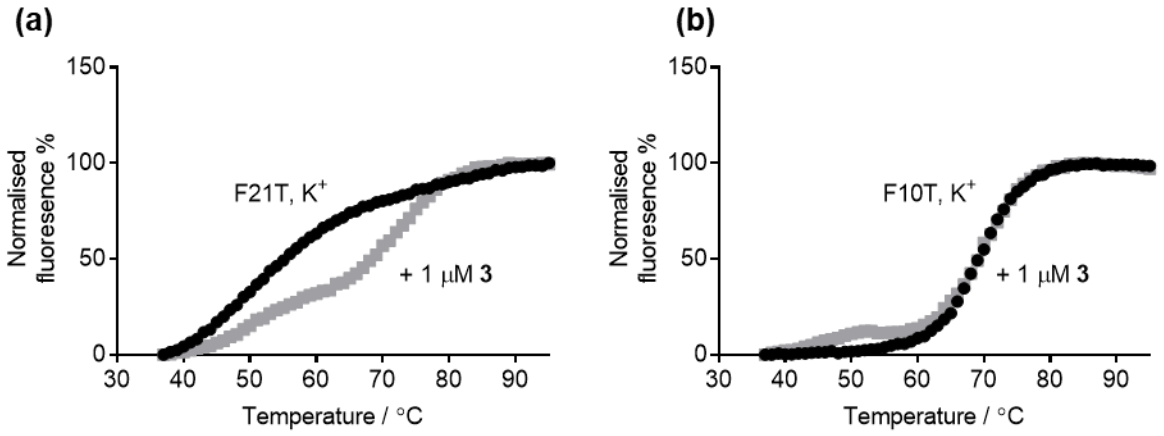

Figure S3: Representative FRET melting curves for ligand **3** against (a) F21T (K^+ form) and (b) F10T. The biphasic melting profile in the presence of ligand may arise from the stabilisation of a partially folded or mis-folded intermediate species by ligand **3** in addition to the fully folded G4 species.[1]

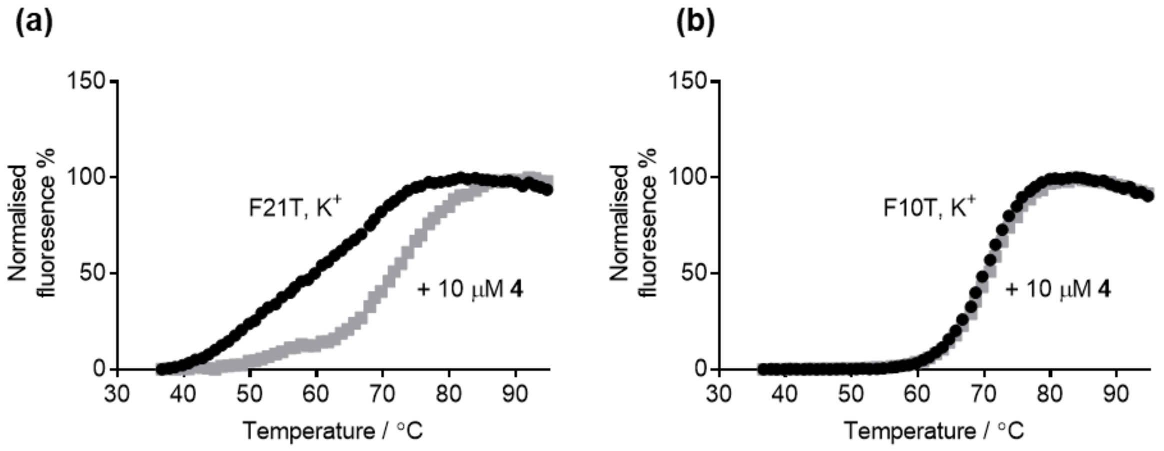

Figure S4: Representative FRET melting curves for ligand **4** against (a) F21T (K^+ form) and (b) F10T.

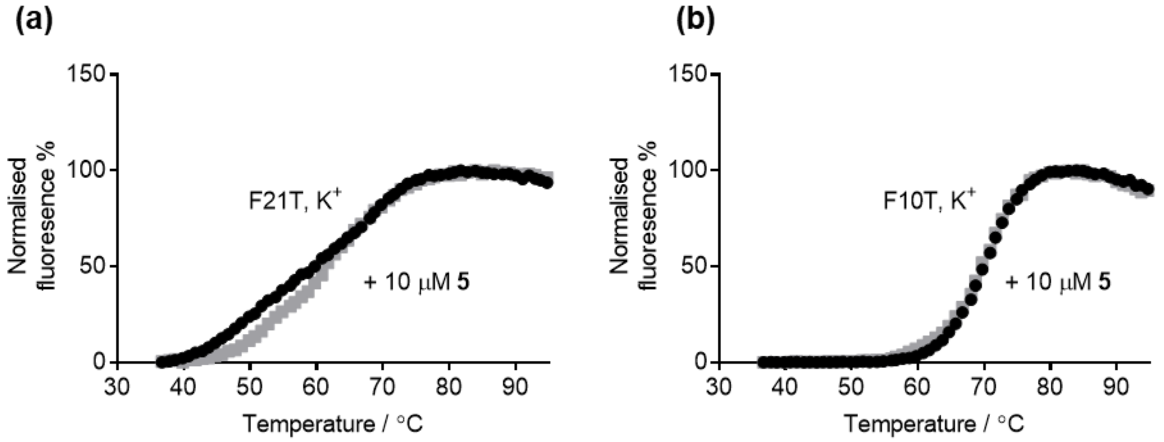

Figure S5: Representative FRET melting curves for ligand **5** against (a) F21T (K^+ form) and (b) F10T.

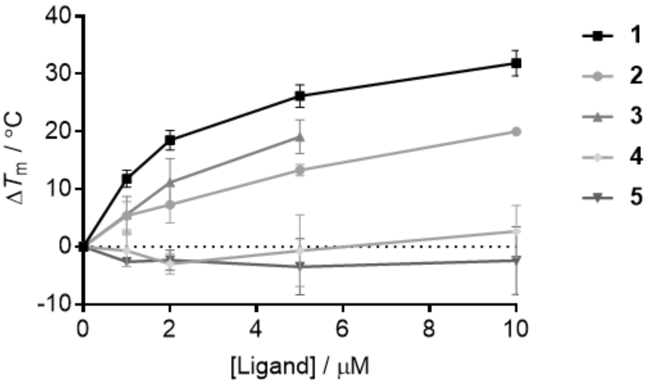

Figure S6: Dependence of ΔT_m of F21T (Na^+ form) on concentration of ligands **1**-**5**. Note: ligand **3** appeared to quench the FRET fluorophores at concentrations above 5µM, precluding determination of ΔT_m at higher concentration.

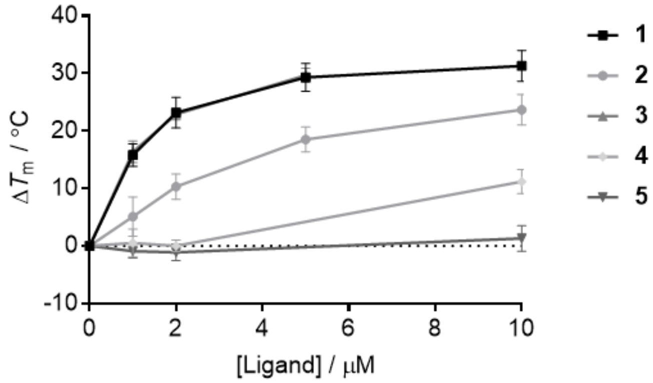

Figure S7: Dependence of ΔT_m of FmycT (K^+ form) on concentration of ligands **1**-**5**. Note: ligand **3** appeared to quench the FRET fluorophores at concentrations above 5µM, precluding determination of ΔT_m at higher concentration. N.B. the curves for ligand **1** and ligand **2** are overlapped.

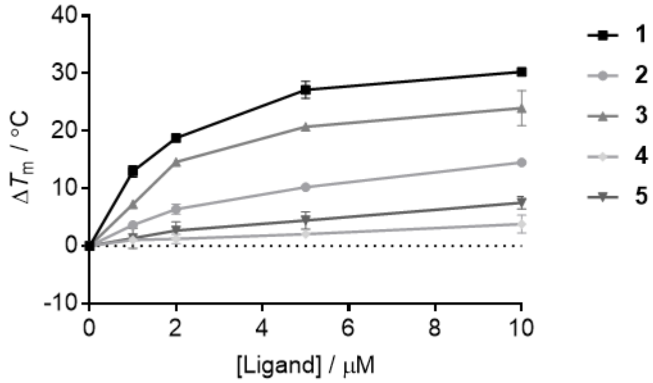

Figure S8: Dependence of ΔT_m of Febr1T (K^+ form) on concentration of ligands **1**-**5**. Note: ligand **3** appeared to quench the FRET fluorophores at concentrations above 5µM, precluding determination of ΔT_m at higher concentration.

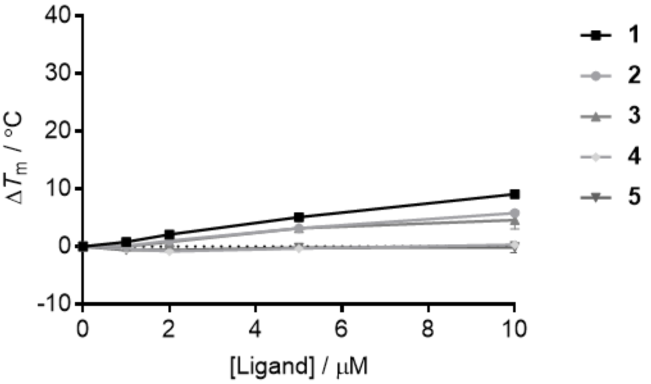

Figure S9: Dependence of ΔT_m of F10T (K^+ form) on concentration of ligands **1**-**5**.

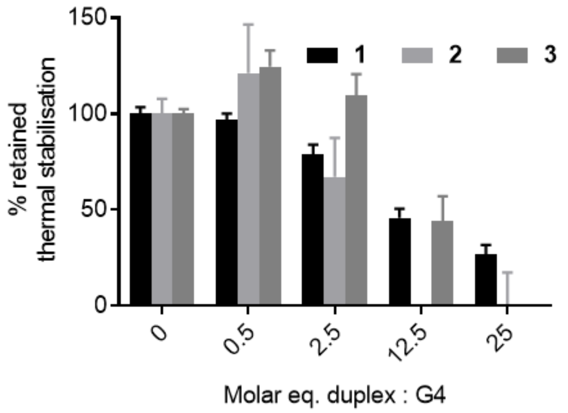

Figure S10: FRET competition assay results for ligands **1**-**3** showing effect on ligand-induced F21T (Na^+) G4 stabilisation in the presence of increasing concentrations of duplex (ds26) DNA competitor. [ligand] = 1 µM, [G4] = 0.2 µM.

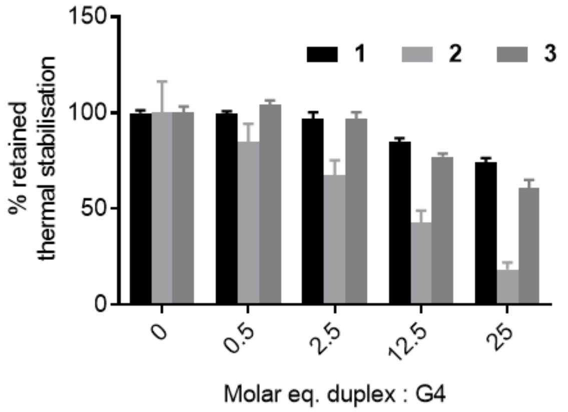

Figure S11: FRET competition assay results for ligands **1**-**3** showing effect on ligand-induced FmycT (K^+) G4 stabilisation in the presence of increasing concentrations of duplex (ds26) DNA competitor. [ligand] = 1 µM, [G4] = 0.2 µM.

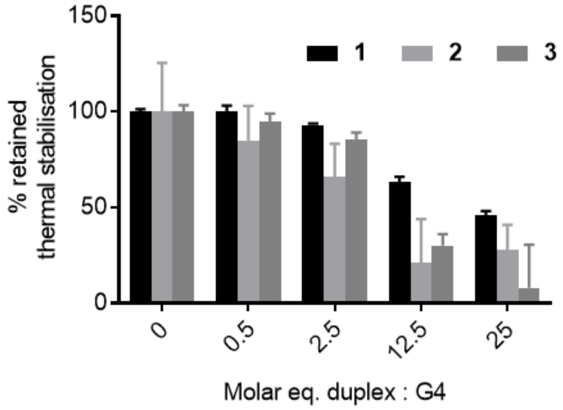

Figure S12: FRET competition assay results for ligands **1**-**3** showing effect on ligand-induced Febr1 (K^+) G4 stabilisation in the presence of increasing concentrations of duplex (ds26) DNA competitor. [ligand] = 1 μM, [G4] = 0.2 μM.

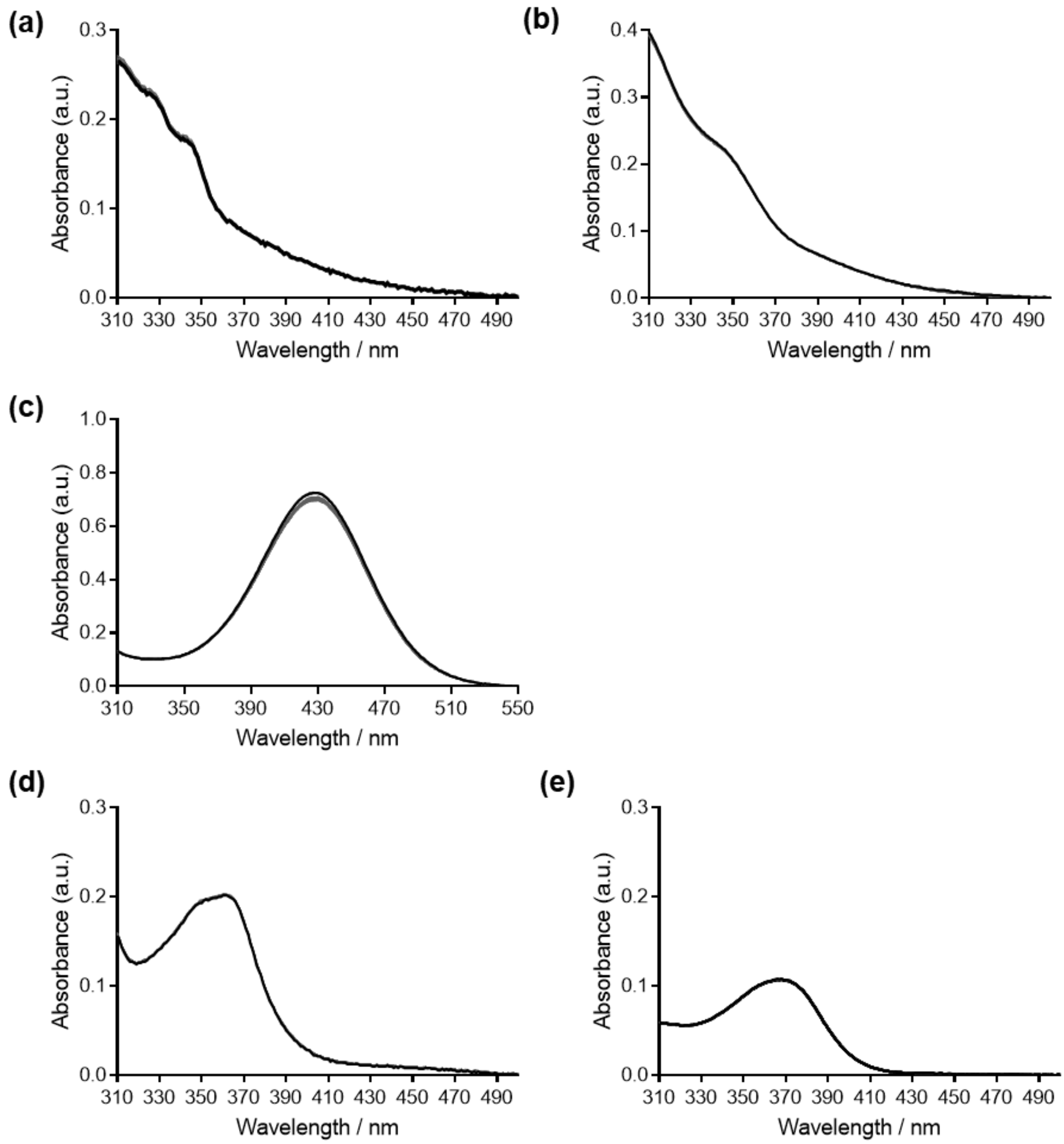

Figure S13: Control experiments to verify the photostability of ligands **1**-**5** to the experimental conditions of spectrophotometric binding studies. The UV-visible spectra of the ligands (10 µM) were recorded in 100 mM potassium phosphate buffer at pH 7.4 up to 18 times (to simulate a spectrophotometric titration experiment). The spectra (superimposed for each ligand in the above figure) remain identical, demonstrating the photostability of the ligands under the experimental conditions.

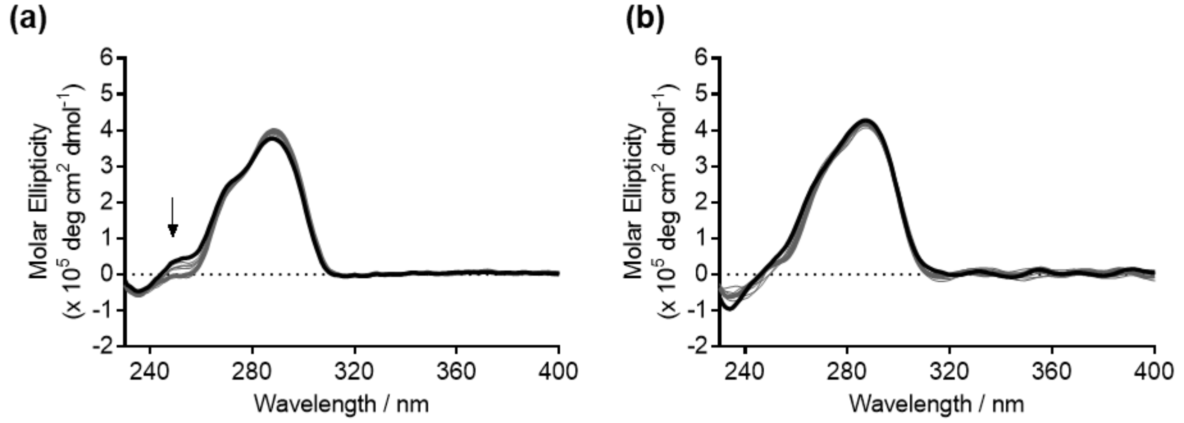

Figure S14: circular dichroism spectra of telo23 G4 (heavy black trace) titrated with 1-7 eq. ligand (a) **4** and (b) **5**. The final titration point is shown as a heavy grey trace, intermediate titration points in light grey traces. See Section 1.2 for full experimental details.

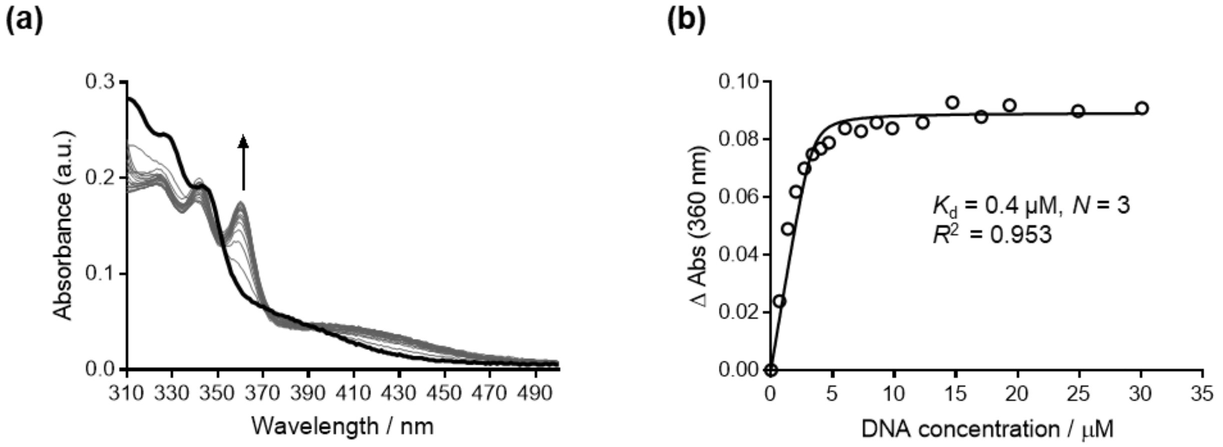

Figure S15: UV-visible titration and determination of apparent binding constant of telo23 G4 and ligand **1**.[8] [**1**] = 10 µM, buffer: 100 mM potassium phosphate, pH 7.4. (a) Raw UV spectra, (b) 360 nm binding isotherm and data fitting parameters. See Section 1.3 for full experimental details.

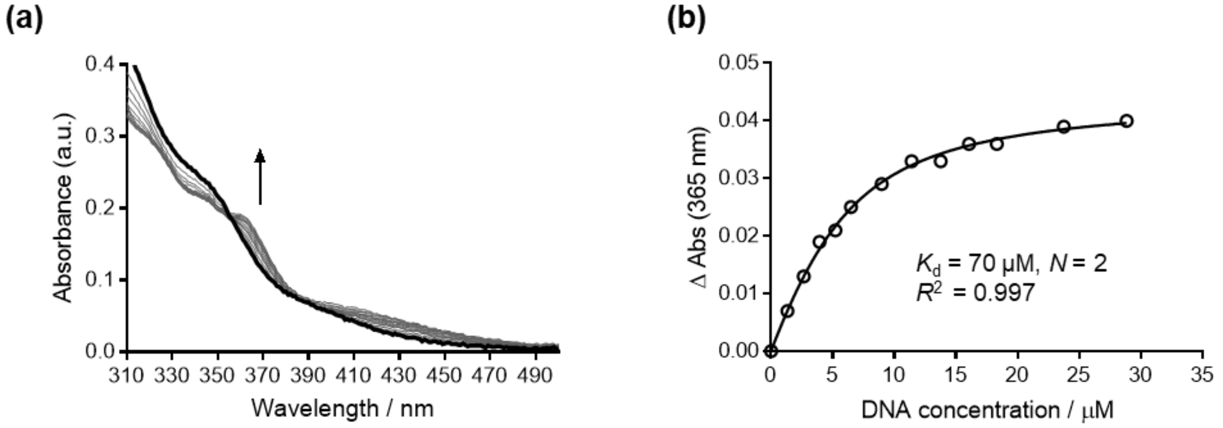

Figure S16: UV-visible titration and determination of apparent binding constant of telo23 G4 and ligand **2**. [**2**] = 10 µM, buffer: 100 mM potassium phosphate, pH 7.4. (a) Raw UV spectra, (b) 365 nm binding isotherm and data fitting parameters. See Section 1.3 for full experimental details.

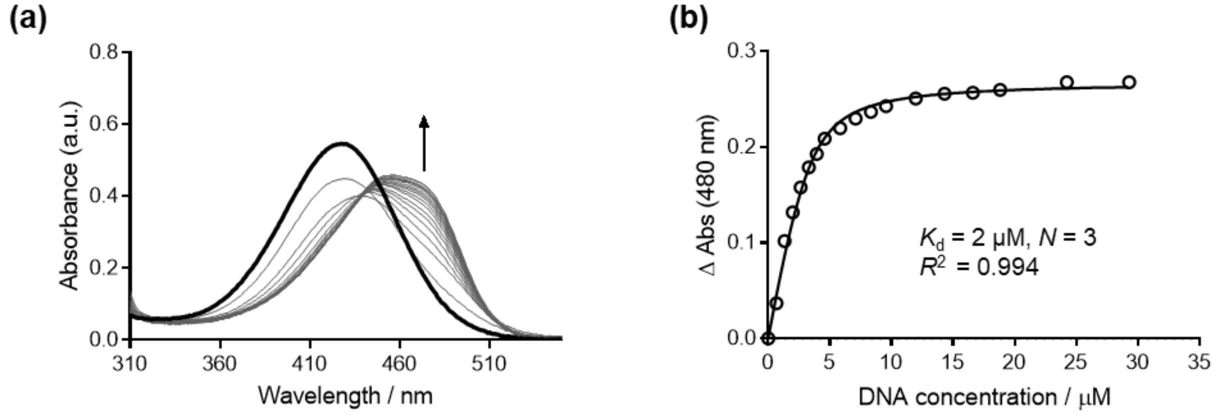

Figure S17: UV-visible titration and determination of apparent binding constant of telo23 G4 and ligand **3**. [**3**] = 10 µM, buffer: 100 mM potassium phosphate, pH 7.4. (a) Raw UV spectra, (b) 480 nm binding isotherm and data fitting parameters. See Section 1.3 for full experimental details.

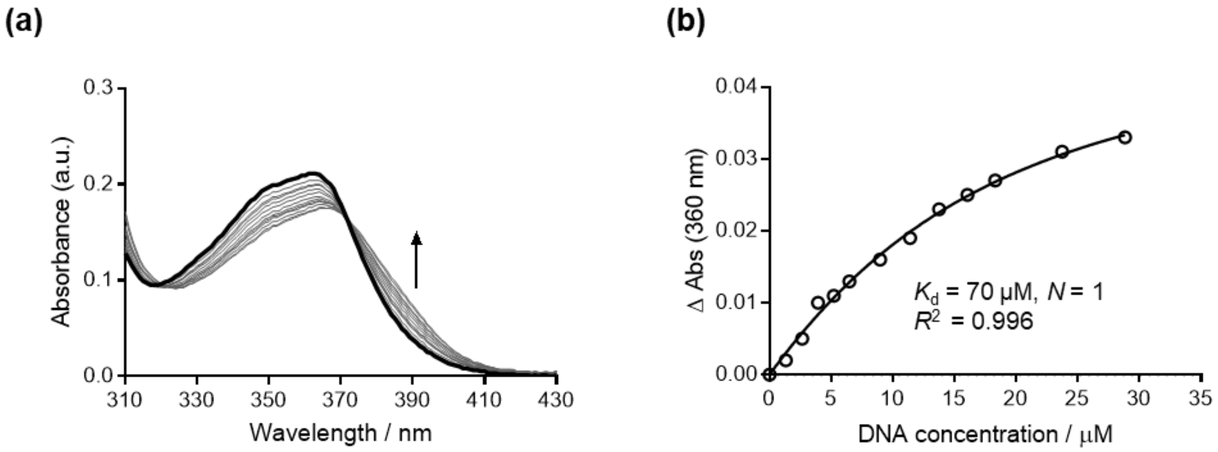

Figure S18: UV-visible titration and determination of apparent binding constant of telo23 G4 and ligand **4**. [**4**] = 10 µM, buffer: 100 mM potassium phosphate, pH 7.4. (a) Raw UV spectra, (b) 380 nm binding isotherm and data fitting parameters. See Section 1.3 for full experimental details.

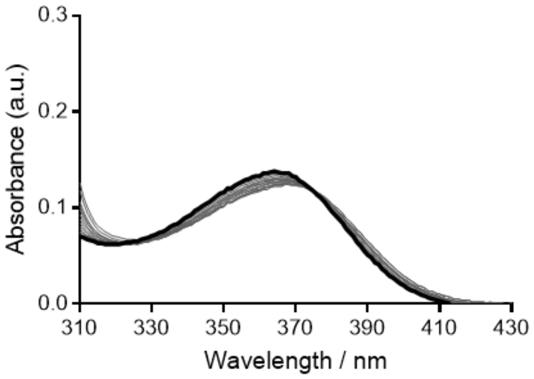

Figure S19: UV-visible titration of telo23 G4 and ligand **5**. [**2**] = 10 µM, buffer: 100 mM potassium phosphate, pH 7.4. Owing to the weak binding of ligand **5**, the binding constant could not be reliably determined.

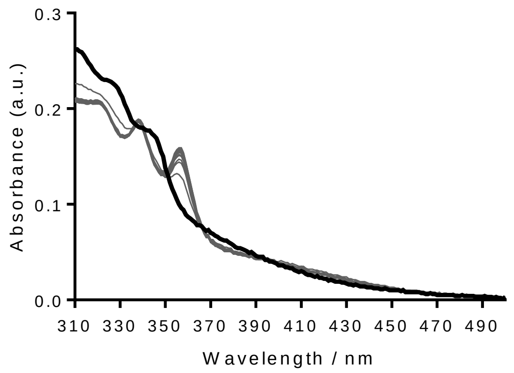

Figure S20: UV-visible titration and determination of apparent binding constant of duplex ds26 DNA and ligand **1**. [**1**] = 10 µM, buffer: 100 mM potassium phosphate, pH 7.4. (a) Raw UV spectra, (b) 360 nm binding isotherm and data fitting parameters. See Section 1.3 for full experimental details.

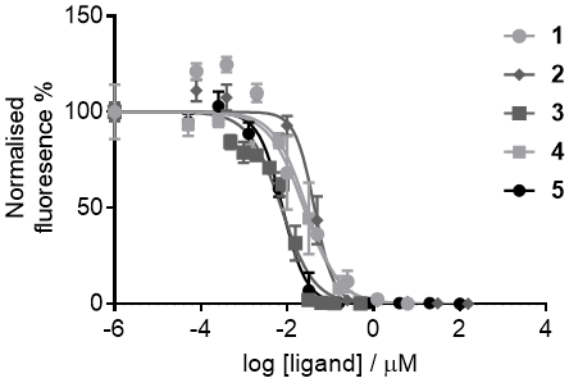

Figure S21: Cell viability dose-response curves for compounds **1**-**5** against *T. brucei* parasites at 72 h exposure. See Section 1.6 for full experimental details.

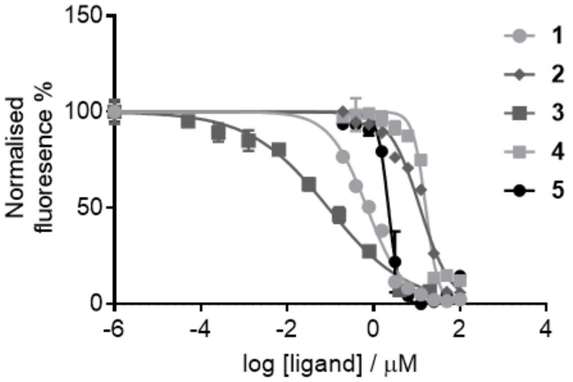

Figure S22: Cell viability dose-response curves for compounds **1**-**5** against *L. major* parasites at 72 h exposure. See Section 1.6 for full experimental details.

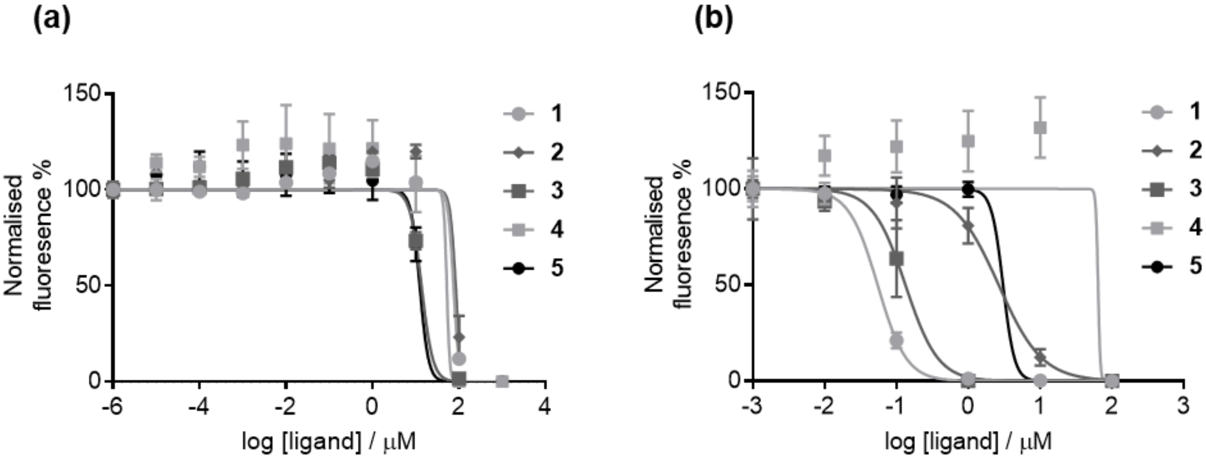

Figure S23: Cell viability dose-response curves for compounds **1**-**5** against HeLa cells at (a) 72 h and (b) 7-day exposure. See Section 1.5 for full experimental details.

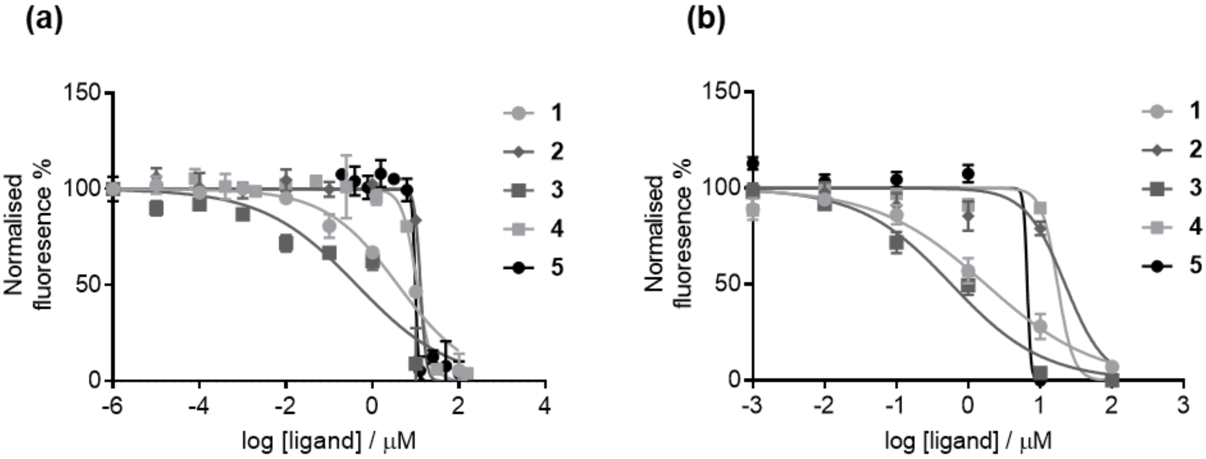

Figure S24: Cell viability dose-response curves for compounds **1**-**5** against MRC-5 cells at (a) 72 h and (b) 7-day exposure. See Section 1.5 for full experimental details.

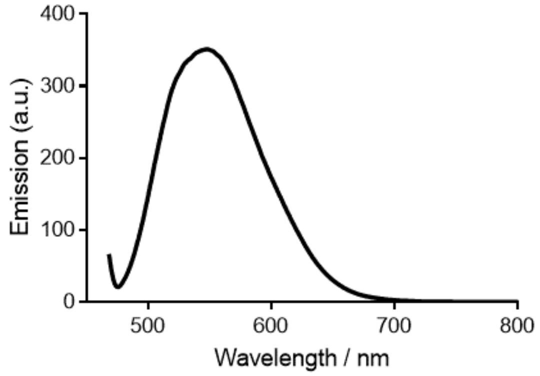

Figure S25: Emission spectrum of ligand **3** in water (0.5% DMSO). [**3**] = 50 µM.

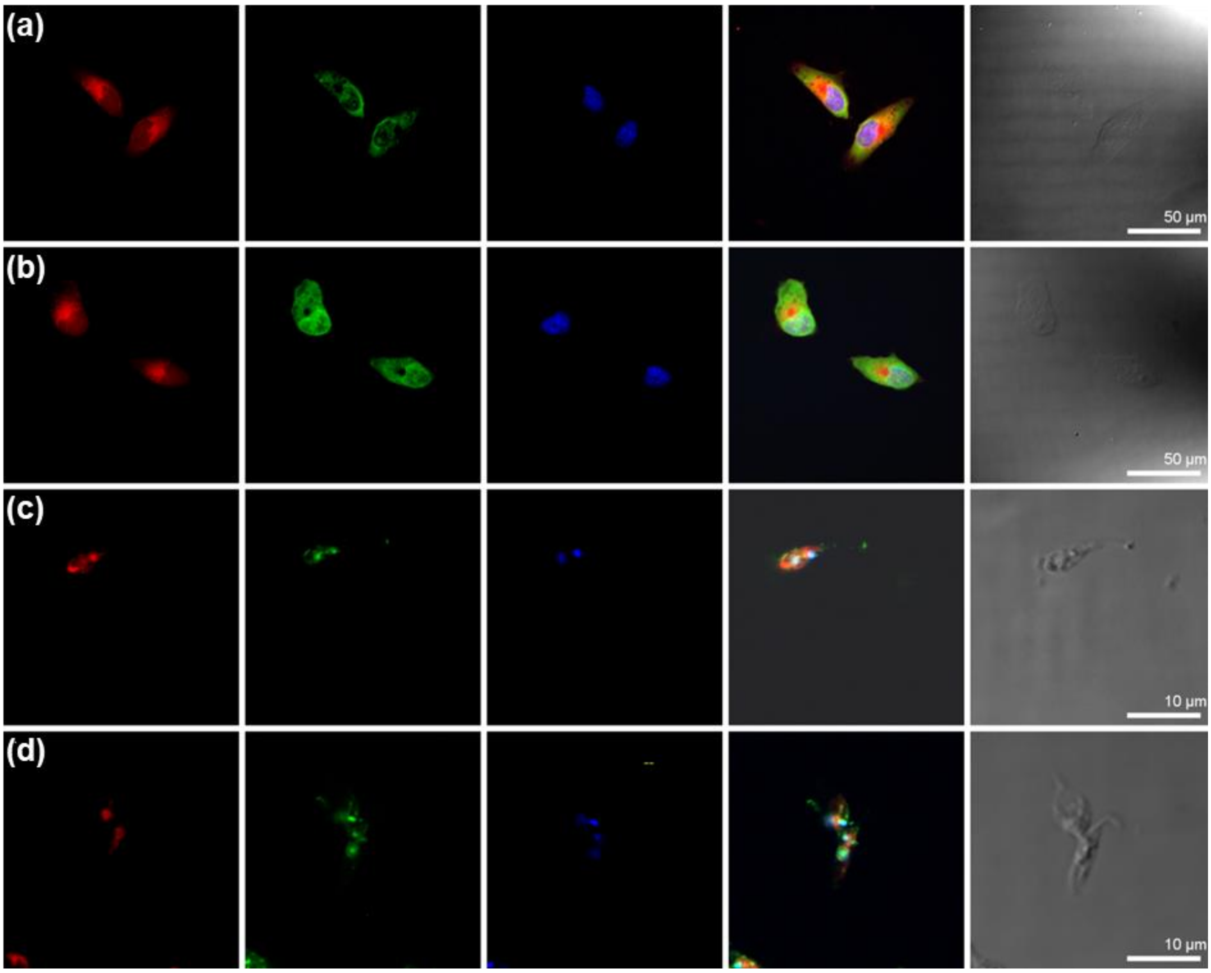

Figure S26: Fluorescence microscopy images of MRC5 non-tumoral cells and *L. major* parasites after incubation with 5 or 1 µM ligand **3**, respectively for 30 min (a, c) or 150 min (b, d). Visualization panes from left to right: mitochondria (Mitotracker Red), ligand **3**, nucleus (DAPI), co-localisation image, bright-field image. Scale bar: 50 µm for cells and 10 µm for parasites.

3 Supplementary tables

Table S1: Viability assay data for ligands **1**-**5** against tumoral (HeLa) cells following 72h incubation.

Ligand	GI_{50} / µM
**1**	77 ± 4
**2**	82 ± 2
**3**	16 ± 3
**4**	46 ± 8
**5**	9.0 ± 3

4 Synthetic procedures and compound characterisation

General experimental

Chemicals were purchased and used without further purification. Dry solvents were obtained by distillation using standard procedures, or by passage through a column of anhydrous alumina using equipment from Anhydrous Engineering (University of Bristol) based on the Grubbs' design.[9] Reactions requiring anhydrous conditions were performed under N_2; glassware and needles were either flame dried immediately prior to use, or placed in an oven (150 °C) for at least 2 h and allowed to cool in a desiccator or under reduced pressure. Liquid reagents, solutions or solvents were added via syringe through rubber septa; solid reagents were added via Schlenk type adapters. Reactions were monitored by TLC on Kieselgel $60F_{254}$ (Merck), with UV light (254 nm) detection and by staining with basic potassium permanganate solution. Flash column chromatography was performed according to Still and co-workers[10] using silica gel [Merck, 230−400 mesh (40−63 µm)]. Solvents for flash column chromatography (FCC) and thin layer chromatography (TLC) are listed in volume:volume percentages. Extracts were concentrated *in vacuo* using both a Heidolph HeiVAP Advantage rotary evaporator (bath temperatures up to 50 °C) at a pressure of 15 mmHg (diaphragm pump) or 0.1 mmHg (oil pump), as appropriate, and a high vacuum line at room temperature. Water soluble compounds were freeze dried on a Lytotrap Plus (LTE Scientific LTD). Preparative HPLC was performed on a Grace Discovery Sciences Reveleris Prep System with a Phenomenex Luna 5 µm C18(2) 100 Å AXIA packed

(250 × 21.2 mm) column. For purification, eluted compounds were detected by UV absorbance at 254 nm. Flow rates were 14 mL / min. The mobile phases used were 0.05 % trifluoroacetic acid in water for the aqueous phase and 0.05 % trifluoroacetic acid in acetonitrile for the organic phase. The gradient was from 5 % organic phase for 5 min the start to 80% organic phase over 20 min before 10 min at 100 % organic phase followed by 5 min at 5 % organic phase. The fractions were combined and concentrated in the same manner as for FCC. ^{1}H NMR and ^{13}C NMR spectra were measured at 25°C in the solvent specified with Varian or Bruker spectrometers operating at field strengths listed. Chemical shifts are quoted in parts per million with spectra referenced to the residual solvent peaks. Multiplicities are abbreviated as: br (broad), s (singlet), d (doublet), t (triplet), q (quartet), p (pentet), m (multiplet) and app. (apparent) or combinations thereof. Assignments of ^{1}H NMR and ^{13}C NMR signals were made where possible, using COSY, HSQC and HMBC experiments. Mass spectra were obtained by the University of Bristol mass spectrometry service by electrospray ionisation (ESI) or matrix assisted laser desorption ionisation (MALDI) modes. Infra-red spectra were recorded in the range 4000-400 cm^{-1} on a Perkin Elmer Spectrum either as neat films or solids compressed onto a diamond window.

(*E*)-6,6'-dibromo-2,2',3,3'-tetrahydro-1,1'-biindenylidene, *m*,(*E*)-7

(*Z*)-6,6'-dibromo-2,2',3,3'-tetrahydro-1,1'-biindenylidene, *m*,(*Z*)-7

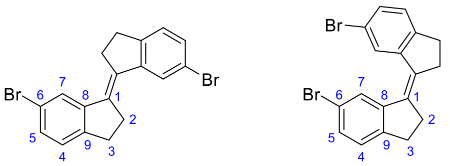

Zinc powder (5.9 g, 90 mmol) was suspended in anhydrous THF (75 mL). $TiCl_4$ (4.9 mL, 45 mmol) was added dropwise as the solution was stirred vigorously. The solution was heated to reflux and stirred for 2 h. The mixture was then cooled to rt and 6-bromo-1-indanone (*m*-**6**, 4.8 g, 23 mmol) was added to the suspension. The mixture was heated to reflux and stirred for 16 h, then quenched with sat. aq. NH_4Cl solution (50 mL) and extracted with $CHCl_3$ (3 × 100 mL). The combined organic extractions were washed with H_2O (100 mL) and then concentrated *in vacuo* to approximately 30 mL, at which volume a precipitate formed. The precipitate was collected by filtration under reduced pressure and dried under vacuum to afford bromide *m*,(*E*)-**7** as a yellow solid (2.4 g, 55%). The filtrate was concentrated *in vacuo* and the residue purified by flash silica chromatography, eluting with hexane, to afford bromide *m*,(*Z*)-**7** as a white solid (1.0 g, 22%).

m,(*E*)-**7**:

^{1}H NMR (500 MHz, DMSO-d$_6$) δ 7.68 (2H, d, *J* = 1.8 Hz, 7-C*H*), 7.41 (2H, dd, *J* = 8.0, 1.8 Hz, 5-C*H*), 7.33 (2H, d, *J* = 8.0 Hz, 4-C*H*), 3.13 – 3.08 (4H, m, 2-C*H*$_2$ or 3-C*H*$_2$), 3.07 – 3.02 (4H, m, 2-C*H*$_2$ or 3-C*H*$_2$).

^{13}**C NMR** Too insoluble for this experiment.

ν$_{max}$ / cm^{-1} (compressed solid) 2925 (w), 2887 (w), 2850 (w), 1889 (w), 1589 (m), 1560 (m), 1464 (m), 1433 (m), 1405 (m), 1205 (m), 1174 (m) 1078 (m), 1037 (m), 879 (s), 819 (s).

EI-LRMS for $C_{18}H_{14}Br_2^+$ [M]$^+$ calcd: 389.9, found: 390.2.

Melting point (toluene) 243 – 246 °C, lit: 240 – 243 °C (solvent not reported).

Proton NMR was consistent with literature data.[11]

m,(*Z*)-**7**:

^{1}**H NMR** (500 MHz, DMSO-d$_6$) δ 8.01 (2H, d, *J* = 1.8 Hz, 7-C*H*), 7.39 (2H, dd, *J* = 8.0, 1.8 Hz, 5-C*H*), 7.32 (2H, d, *J* = 8.0 Hz, 4-C*H*), 2.93 – 2.90 (4H, m, 2-C*H*$_2$ or 3-C*H*$_2$), 2.80 – 2.76 (4H, m, 2-C*H*$_2$ or 3-C*H*$_2$).

^{13}**C NMR** (126 MHz, DMSO-d$_6$) δ 147.6 (9-*C*), 141.8 (8-*C*), 135.2 (1-*C*), 130.2 (7-CH), 127.6, 125.0 (4-*C*H and 5-*C*H), 118.7 (6-*C*), 34.4 (2-*C*H or 3-*C*H), 29.6 (2-*C*H or 3-*C*H).

ν$_{max}$ / cm^{-1} (compressed solid) 2029 (w), 2895 (w), 2871 (w), 2837 (w), 1635 (w), 1590 (m), 1559 (m), 1459 (m), 1444 (m), 1425 (m), 1264 (m), 1166 (m), 1071 (m), 803 (s).

EI-LRMS for $C_{18}H_{14}Br_2^+$ [M]$^+$ calcd: 389.9, found: 390.0.

Melting point (toluene) 146 – 147 °C, lit: 138 – 140 °C (solvent not reported).

Proton and carbon NMR were consistent with literature data.[11]

(*E*)-5,5'-dibromo-2,2',3,3'-tetrahydro-1,1'-biindenylidene, *p*,(*E*)-7

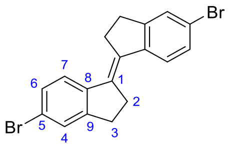

Zinc powder (3.9 g, 60 mmol) was suspended in anhydrous THF (30 mL). $TiCl_4$ (3.3 mL, 30 mmol) was added dropwise as the solution was stirred vigorously. The solution was heated to reflux and stirred for 2 h. The mixture was then cooled to rt and 5-bromo-1-indanone (*p*-**6**, 3.2 g, 15 mmol) was added to the suspension. The mixture was heated to reflux and stirred for 30 h, then quenched with sat. aq. NH_4Cl solution (50 mL) and extracted with $CHCl_3$ (3 × 100 mL). The combined organic extractions were washed with H_2O (100 mL) and then concentrated *in vacuo* to approximately 50 mL, at which volume a precipitate formed. The precipitate was collected by filtration under reduced pressure and dried under vacuum to afford the title compound as an off-white solid (2.0 g, 68%).

^{1}**H NMR** (500 MHz, DMSO-d$_6$) δ 7.55 (2H, s, 4-C*H*), 7.51 (2H, d, *J* = 8.4 Hz, 6-C*H* or 7-C*H*), 7.44 (2H, d, *J* = 8.4 Hz, 6-C*H* or 7-C*H*), 3.08 (8H, app. s, 2-C*H*$_2$ and 3-C*H*$_2$).

^{13}**C NMR** (126 MHz, DMSO-d$_6$) δ 149.7 (9-*C*), 141.6 (8-*C*), 134.8 (1-*C*), 129.4 (6-*C*H or 7-*C*H), 127.9 (4-*C*H), 125.9 (6-*C*H or 7-*C*H), 120.3 (5-*C*), 31.3 (2-*C*H$_2$ or 3-*C*H$_2$), 30.3 (2-*C*H$_2$ or 3-*C*H$_2$).

ν_{max} / cm^{-1} (compressed solid) 2942 (w), 2922 (m), 2877 (w), 2845 (w), 2832 (w), 1853 (w), 1743 (w), 1582 (m), 1561 (w), 1466 (s), 1450 (m), 1422 (m), 1408 (m), 1368 (w), 1346 (w), 1321 (w), 1297 (m), 1280 (m), 1250 (m), 1223 (w), 1204 (m), 1711 (s), 1116 (w), 1070 (m), 871 (s), 819 (m), 783 (s), 653 (m), 551 (s), 428 (s).

MALDI-HRMS for $C_{18}H_{14}Br_2^+$ [M]$^+$ calcd: 389.9442, found: 389.9444.

Melting point (toluene) 269 – 271 °C.

(*E*)-6,6'-di(pyridin-4-yl)-2,2',3,3'-tetrahydro-1,1'-biindenylidene, *m*,(*E*)-9

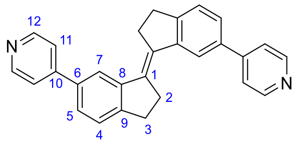

Bromide *m*,(*E*)-**7** (250 mg, 0.63 mmol) and 4-pyridinylboronic acid (**8**, 230 mg, 1.9 mmol) were dissolved in toluene (22.5 mL) and EtOH (2.5 mL) and 2M aq. Na_2CO_3 (5 mL) was added. The solution was degassed by bubbling nitrogen for 30 min. $Pd(PPh_3)_4$ (140 mg, 0.12 mmol) was added in one portion and the mixture degassed for a further 10 min. The reaction was heated to 100 °C and stirred for 16 h. The mixture was then cooled to rt, diluted with CH_2Cl_2 (150 mL) and washed with H_2O (100 mL). The aqueous was back extracted with CH_2Cl_2 (50 mL) and the combined organic extractions dried ($MgSO_4$) and filtered. The filtrate was concentrated *in vacuo* and the residue purified by flash silica chromatography, eluting with 0 – 5% MeOH in CH_2Cl_2, to afford an off-white solid. This was triturated in Et_2O (30 mL) and the remaining insoluble solid filtered and air-dried to afford compound *m*,(*E)*-**9** as an off-white powder (130 mg, 53%).

^{1}H NMR (500 MHz, $CDCl_3$) δ 8.68 (4H, d, *J* = 6.2 Hz, 12-C*H*), 7.86 (2H, s, 7-C*H*), 7.56 (4H, d, *J* = 6.2 Hz, 11-C*H*), 7.50 (2H, d, *J* = 7.8 Hz, 5-C*H*), 7.46 (2H, d, *J* = 7.8 Hz, 4-C*H*), 3.34 – 3.29 (4H, br, 2-CH_2), 3.24 – 3.20 (4H, br, 3-CH_2).

^{13}C NMR (126 MHz, $CDCl_3$) δ 150.4 (12-*C*H), 149.1 (10-*C*), 148.5 (9-*C*), 144.2 (8-*C*), 136.9 (6-*C*), 135.9 (1-*C*), 126.3 (5-*C*H), 125.8 (4-*C*H), 123.2 (7-*C*H), 121.9 (11-*C*H), 32.4 (2-*C*H_2), 31.1 (3-*C*H_2).

ν_{max} / cm^{-1} (compressed solid) 3025 (w) 2942 (w), 2929 (w), 2911 (w), 2845 (w), 1593 (s), 1542 (m), 1474 (s), 1453 (w) 1434 (w), 1422 (w), 1440 (m), 1270 (m), 1069 (m) 992 (m), 887 (w), 839 (m), 812 (s), 800 (s), 729 (m), 607 (m), 535 (s).

ESI-HRMS for $C_{28}H_{23}N_2^+$ [MH]$^+$ calcd: 387.1856, found: 387.1871.

Melting point (dichloromethane) Decomp. > 170 °C.

(*Z*)-6,6'-di(pyridin-4-yl)-2,2',3,3'-tetrahydro-1,1'-biindenylidene, *m*,(*Z*)-9

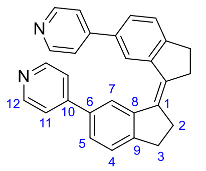

Bromide *m*,(*Z*)-**7** (500 mg, 1.3 mmol) and 4-pyridinylboronic acid (**8**, 500 mg, 4.0 mmol) were dissolved in toluene (23 mL) and EtOH (2.5 mL) and 2M aq. Na_2CO_3 (5.0 mL) was added. The solution was degassed by bubbling a stream of nitrogen for 30 min. $Pd(PPh_3)_4$ (150 mg, 0.13 mmol) was added in one portion and the mixture degassed for a further 10 min. The reaction was heated to 100 °C and stirred for 16 h. The mixture was then cooled to rt, diluted with CH_2Cl_2 (150 mL) and washed with H_2O (100 mL). The aqueous layer was back extracted with CH_2Cl_2 (50 mL) and the combined organic extractions dried ($MgSO_4$) and filtered. The filtrate was concentrated *in vacuo* and the residue purified by flash silica chromatography, eluting with 0 – 5% MeOH in CH_2Cl_2, to afford a white solid. This was triturated in Et_2O (30 mL) and the remaining insoluble solid filtered and air-dried to afford *m*,(*Z*)-**9** compound as an off-white powder (260 mg, 52%).

^{1}H NMR (500 MHz, $CDCl_3$) δ 8.45 (4H, dd, *J* = 4.5, 1.8 Hz, 12-C*H*), 8.39 (2H, app. s, 7-C*H*), 7.44 (2H, dd, *J* = 7.8, 1.6 Hz, 5-C*H*), 7.41 (2H, d, *J* = 7.8 Hz, 4-C*H*), 7.29 (4H, dd, 4.5, 1.8 Hz, 11-C*H*), 3.09 – 3.05 (4H, m, 3-C*H*$_2$), 2.92 – 2.89 (4H, m, 2-C*H*$_2$).

^{13}C NMR (126 MHz, $CDCl_3$) δ 150.3 (12-*C*H), 149.6 (9-*C*), 148.7 (10-*C*), 141.5 (8-*C*), 136.0 (6-*C*), 135.4 (1-*C*), 126.4 (5-*C*H), 126.2 (4-*C*H), 122.1 (7-*C*H), 121.6 (11-*C*H), 34.9 (2-*C*H), 30.6 (3-*C*H).

ν_{max} / cm^{-1} (compressed solid) 3066 (w), 3027 (w), 2939 (w), 2905 (w), 2840 (w), 1596 (s), 1543 (m), 1472 (m), 1446 (w), 1422 (w), 1406 (w), 1268 (w), 1220 (w), 992 (w), 809 (s), 730 (m), 613 (w), 606 (w), 533 (w).

ESI-HRMS for $C_{28}H_{23}N_2^+$ (MH^+) calcd: 387.1856, found 387.1841.

Melting point (dichloromethane) Decomp. > 170 °C.

(*E*)-5,5'-di(pyridin-4-yl)-2,2',3,3'-tetrahydro-1,1'-biindenylidene, *p*,(*E*)-9

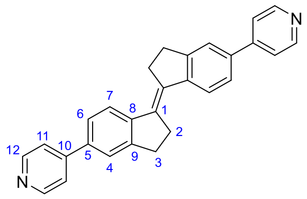

Bromide *p*,(*E*)-**7** (250 mg, 0.64 mmol) and 4-pyridinylboronic acid (**8**, 240 mg, 1.9 mmol) were dissolved in toluene (23 mL) and EtOH (2.5 mL) and 2M aq. Na_2CO_3 (5.0 mL) was added. The solution was degassed by bubbling nitrogen for 30 min. $Pd(PPh_3)_4$ (74 mg, 0.064 mmol) was added in one portion and the mixture degassed for a further 10 min. The reaction was heated to 100 °C and stirred for 16 h. The mixture was then cooled to rt, diluted with CH_2Cl_2 (150 mL) and washed with H_2O (100 mL). The aqueous was back extracted

with CH_2Cl_2 (50 mL) and the combined organic extractions dried ($MgSO_4$) and filtered. The filtrate was concentrated *in vacuo* and the residue purified by flash silica chromatography, eluting with 0 – 5% MeOH in CH_2Cl_2, to afford *p*,(*E*)-**9** as a yellow solid (190 mg, 76%).

^{1}H NMR (400 MHz, $CDCl_3$) δ 8.66 (4H, d, *J* = 6.2 Hz, 12-C*H*), 7.72 (2H, d, *J* = 8.2 Hz, 7-C*H*), 7.62 (2H, d, *J* = 1.7 Hz, 4-C*H*), 7.56 (2H, dd, *J* = 8.2, 1.7 Hz, 6-C*H*), 7.54 (4H, d, *J* = 6.2 Hz, 11-C*H*), 3.24 (8H, app. s, 2-CH_2 and 3-CH_2).

^{13}C NMR (101 MHz, $CDCl_3$) δ 150.4 (12-*C*H), 148.5 (9-*C*), 148.2 (10-*C*), 144.2 (8-*C*), 136.9 (5-*C*), 136.2 (1-*C*), 125.6 (6-*C*H), 125.2 (7-*C*H), 123.6 (4-*C*H), 121.6 (11-*C*H), 32.2 (2-CH_2), 31.2 (3-CH_2).

ν_{max} / cm^{-1} (compressed solid) 3485 (w), 3027 (w), 2920 (w), 2844 (w), 1594 (s), 1539 (m), 1476 (m), 1428 (w), 1407 (m), 1308 (w), 1298 (w), 1258 (w), 1221 (w), 997 (w), 888 (w), 809 (s), 731 (m), 719 (m), 613 (w), 527 (w).

ESI-HRMS for $C_{28}H_{23}N_2^+$ [MH]$^+$ calcd: 387.1856, found: 387.1872.

Melting point (chloroform) Decomp. > 150 °C.

(*E*)-4,4'-(2,2',3,3'-tetrahydro-[1,1'-biindenylidene]-6,6'-diyl)bis(1-methylpyridin-1-ium) iodide, 1

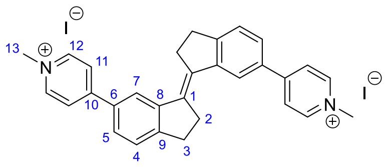

Pyridine *m*,(*E*)-**9** (40 mg, 0.10 mmol), was dissolved in anhydrous DMF (6.0 mL) and the solution heated to 90 °C. Methyl iodide (15 µL, 0.24 mmol) was added and the reaction stirred for 1 h. The mixture was then cooled to rt and concentrated *in vacuo.* The residue was suspended acetone (5.0 mL), filtered and the dark solid air dried to afford compound **1** (15 mg, 35%).

^{1}H NMR (500 MHz, DMSO-d$_6$) δ 9.00 (4H, d, *J* = 6.9 Hz, 12-C*H*), 8.56 (4H, d, *J* = 6.9 Hz, 11-C*H*), 8.11 (2H, d, *J* = 1.6 Hz, 7-C*H*), 7.95 (2H, dd, *J* = 7.9, 1.6 Hz, 5-C*H*), 7.66 (2H, d, *J* = 7.9 Hz, 4-C*H*), 4.36 (6H, s, 13-CH_3), 3.40 – 3.36 (4H, m, 2-CH_2), 3.25 – 3.22 (4H, m, 3-CH_2).

^{13}C NMR (126 MHz, DMSO-d$_6$) δ 154.8 (10-*C*), 151.6 (9-*C*), 145.5 (12-*C*H), 143.8 (8-*C*), 135.7 (1-*C*), 132.4 (6-CH_2), 127.6 (5-*C*H), 126.4 (4-*C*H), 124.3 (11-*C*H), 123.6 (7-*C*H), 47.1 (13-CH_3), 31.4 (2-CH_2), 30.7 (3-CH_2).

ν_{max} / cm^{-1} (compressed solid) 3121 (w), 3088 (w), 3038 (w), 2930 (w), 2910 (w), 2844 (w), 1636 (s), 1601 (s), 1558 (m), 1522 (m), 1472 (s), 1439 (m), 1414 (m), 1348 (w), 1312 (m) 1298 (m), 1289 (m), 1272 (m), 1223 (w), 1198 (s), 856 (w), 820 (s), 800 (m), 723 (m), 607 (m), 587 (m), 534 (m), 510 (s), 426 (s).

MALDI-HRMS $C_{29}H_{25}N_2^+$ [M-CH_3]$^+$ calcd: 401.2012, found 401.2019.

Melting point Decomp. > 210 °C.

(*Z*)-4,4'-(2,2',3,3'-tetrahydro-[1,1'-biindenylidene]-6,6'-diyl)bis(1-methylpyridin-1-ium) iodide, 2

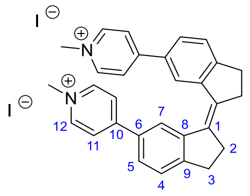

Pyridine *m*,(*Z*)-**9** (22 mg, 0.57 mmol) was dissolved in anhydrous DMF (3.0 mL) and the solution heated to 90 °C. Methyl iodide (8.0 µL, 0.12 mmol) was added and the reaction stirred for 1 h. The mixture was then cooled to rt and concentrated *in vacuo.* The residue was suspended acetone (5.0 mL), filtered and the dark solid air dried to afford the title compound (15 mg, 40%).

^{1}H NMR (500 MHz, DMSO-d_6) δ 8.82 (4H, d, *J* = 6.5 Hz, 12-C*H*), 8.64 (2H, d, *J* = 1.9 Hz, 7-C*H*), 8.31 (4H, d, *J* = 6.5 Hz, 11-C*H*), 7.97 (2H, dd, *J* = 7.9, 1.9 Hz, 5-C*H*), 7.66 (2H, d, *J* = 7.9 Hz, 4-C*H*), 4.28 (6H, s, 13-CH_3), 3.13 – 3.08 (4H, m, 3-CH_2), 2.95 – 2.92 (4H, m, 2-CH_2).

^{13}C NMR (126 MHz, DMSO-d_6) δ 154.3 (10-*C*), 153.0 (9-*C*), 145.4 (12-*C*H), 141.1 (8-*C*), 135.5 (1-*C*), 131.4 (6-*C*), 127.8 (5-*C*H), 126.9 (4-*C*H), 123.5 (11-*C*H), 121.9 (7-*C*H), 47.0 (13-CH_3), 34.5 (2-CH_2), 30.1 (3-CH_2).

ν_{max} / cm^{-1} (compressed solid) 3015 (w), 2929 (w), 1639 (s), 1594 (m), 1558 (m), 1522 (m), 1473 (m), 1443 (m), 1425 (m), 1321 (m), 1279 (m), 1203 (m), 1194 (m), 819 (S), 722 (w), 599 (m), 508 (s), 425 (s).

ESI-HRMS $C_{30}H_{28}N_2^{2+}$ [M]$^{2+}$ calcd: 208.1121, found 208.1125.

Melting point Decomp. > 210 °C.

(*E*)-4,4'-(2,2',3,3'-tetrahydro-[1,1'-biindenylidene]-5,5'-diyl)bis(1-methylpyridin-1-ium) iodide, 3

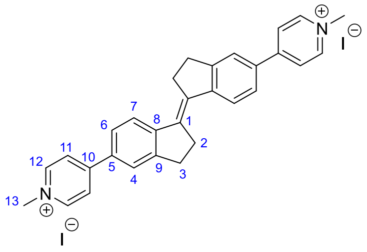

Pyridine *p*,(*E*)-**9** (50 mg, 0.13 mmol), was dissolved in anhydrous DMF (2.0 mL) and the solution heated to 90 °C. Methyl iodide (24 µL, 0.34 mmol) was added and the reaction stirred for 1 h. The mixture was then cooled to rt and concentrated *in vacuo.* The residue was suspended in 1:1 H_2O/MeCN (5.0 mL) and the precipitate filtered and dried under vacuum to afford the title compound as a bright orange powder (47 mg, 55%).

^{1}H NMR (500 MHz, DMSO-d_6) δ 8.99 (4H, d, *J* = 6.4 Hz, 12-C*H*), 8.54 (4H, d, *J* = 6.4 Hz, 11-C*H*), 8.15 (2H, s, 4-C*H*), 8.05 (2H, d, *J* = 8.5 Hz, 6-C*H*), 7.87 (2H, d, *J* = 8.5 Hz, 7-C*H*), 4.32 (6H, s, 15-CH_3) 3.33 (4H, s, 2-CH_2), 3.27 (4H, s, 3-CH_2).

^{13}C NMR (126 MHz, DMSO-d_6) δ 154.1 (10-*C*), 149.4 (9-*C*), 146.4 (8-*C*), 145.9 (12-*C*H), 137.9 (1-*C*), 132.7 (5-*C*), 127.3 (6-*C*H), 125.9 (7-*C*H), 125.0 (4-*C*H), 124.1 (11-*C*H), 47.4 (13-CH_3), 32.1 (2-CH_2), 30.9 (3-CH_2).

ν_{max} **/ cm^{-1}** (compressed solid) 3020 (w), 2921 (w), 2882 (w), 1637 (s), 1600 (s), 1574 (m), 1556 (m), 1525 (s), 1485 (s), 1473 (m), 1448 (m), 1422 (m), 1352 (m), 1316 (m), 1306 (m), 1285 (m), 1239 (m), 1191 (s), 1161 (m), 1122 (m), 1053 (m), 822 (m), 849 (m), 829 (s), 795 (s), 714 (m), 666 (m), 508 (s).

ESI-HRMS $C_{29}H_{25}N_2^+$ $[M-CH_3]^+$ calcd: 401.2012, found 401.2018.

Melting point Decomp. > 250 °C.

(*E*)-N6,N6'-bis(3-(4-methylpiperazin-1-yl)propyl)-2,2',3,3'-tetrahydro-[1,1'-biindenylidene]-6,6'-diamine, 4

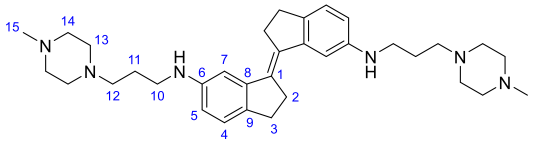

Bromide *m*,(*E*)-**7** (49 mg, 0.13 mmol), BrettPhos Pd G3 (7.0 mg, 7.7 µmol) and sodium *tert*-butoxide (49 mg, 0.51 mmol) were added to an oven-dried microwave vial. The vessel was sealed with a fitted septum and evacuated under vacuum and purged with nitrogen three times. Anhydrous dioxane (2.0 mL) was add and the solution degassed by bubbling nitrogen through the solution for 10 min. 1-(3-Aminoproyl)-4-methylpiperazine (49 µL, 0.29 mmol) was then added and the resulting was stirred at 80 °C for 2 h. The reaction mixture was cooled, concentrated *in vacuo* and the residue suspended in H_2O (5.0 mL). Trifluoroacetic acid (200 µL) was added and the resulting mixture filtered through Celite and purified by reverse phase HPLC to yield the trifluoroacetate salt of (*E*)-**9** as amorphous pale solid (73 mg, 47%).

^{1}H NMR (400 MHz, D_2O) δ 7.67 (2H, d, *J* = 1.6 Hz, 7-C*H*), 7.56 (2H, d, *J* = 8.1 Hz, 4-C*H*), 7.33 (2H, dd, *J* = 8.1, 1.6 Hz, 5-C*H*), 3.79 – 3.46 (16H, br, 13-CH_2 and 14-CH_2), 3.58 (4H, t, *J* = 7.7 Hz, 10-CH_2), 3.34 (4H, t, *J* = 8.0 Hz, 12-CH_2), 3.20 (8H, app. s, 2-CH_2 and 3-CH_2), 3.02 (6H, s, 15-CH_3), 2.30 – 2.20 (4H, m, 11-CH_2).

^{13}C NMR (126 MHz, D_2O) δ 162.87 (q, *J* = 35.5 Hz, CF_3*C*O), 149.46 (9-*C*), 144.17 (8-*C*), 136.32 (1-*C*), 133.15 (6-*C*), 126.69 (4-*C*H), 121.04 (5-*C*H), 118.02 (7-*C*H), 116.28 (q, *J* = 291.8 Hz, *C*F_3CO), 53.45 (12-*C*H_2), 50.37 (14-*C*H_2), 48.86 (13-*C*H_2), 48.13 (10-*C*H_2), 42.75 (15-*C*H_3), 31.49 (2-*C*H_2), 30.18 (3-*C*H_2), 20.59 (11-*C*H_2).

ν_{max} **/ cm^{-1}** 1670 (s), 1460 (m) 1418 (m), 1176 (s), 1120 (s), 961 (m), 831 (s), 797 (s), 720 (s).

ESI-HRMS for $C_{34}H_{51}N_6^+$ $[MH]^+$ calcd: 543.4170, found 543.4163.

Melting point Decomp. > 190 °C.

(*Z*)-*N*^{6},*N*$^{6'}$-bis(3-(4-methylpiperazin-1-yl)propyl)-2,2',3,3'-tetrahydro-[1,1'-biindenylidene]-6,6'-diamine, 5

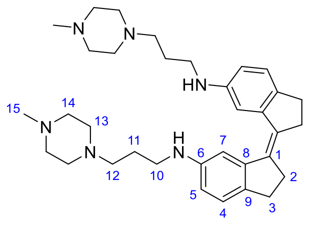

Bromide *p*,(*Z*)-**7** (59 mg, 0.15 mmol), BrettPhos Pd G3 (6.0 mg, 6.6 µmol) and sodium *tert*-butoxide (58 mg, 0.61 mmol) were added to an oven-dried microwave vial. The vessel was sealed with a fitted septum and evacuated under vacuum and purged with nitrogen three times. Anhydrous dioxane (2.0 mL) was add and the solution degassed by bubbling nitrogen through the solution for 10 min. 1-(3-Aminoproyl)-4-methylpiperazine (44 µL, 0.33 mmol) was added and the resulting solution was stirred at 80 °C for 2 h. The reaction mixture was cooled, concentrated *in vacuo* and the residue suspended in H_2O (5.0 mL). Trifluoroacetic acid (200 µL) was added and the resulting mixture filtered through Celite and purified by reverse phase HPLC to yield the trifluoroacetate salt of (*Z*)-**9** as amorphous pale solid (35 mg, 19%).

^{1}H NMR (400 MHz, D_2O) δ 7.95 (2H, s, 7-C*H*), 7.56 (2H, d, *J* = 8.0 Hz, 4-C*H*), 7.29 (2H, d, *J* = 8.0 Hz, 5-C*H*), 3.91 – 3.18 (16H, br, 13-CH_2 and 14-CH_2), 3.47 (4H, t, *J* = 7.9 Hz, 10-CH_2), 3.27 (4H, t, *J* = 8.1 Hz, 12-CH_2), 3.07 – 3.01 (4H, m, 2-CH_2 or 3-CH_2), 3.00 (s, 6H, 15-CH_3) 2.93 – 2.87 (4H, m, 2-CH_2 or 3-CH_2), 2.14 – 2.04 (4H, m, 4H, 11-CH_2).

^{13}C NMR (126 MHz, D_2O) δ 162.9 (q, *J* = 35.4, CF_3CO), 149.4 (9-*C*), 141.5 (8-*C*), 136.0 (1-*C*), 133.8 (6-*C*), 127.2 (4-*C*), 120.5, (5-*C*H), 116.3 (q, *J* = 291.7 Hz, CF_3CO), 115.6 (7-*C*H), 53.5 (12-CH_2), 50.8 (14-CH_2), 48.9 (13-CH_2), 47.2 (10-CH_2), 42.7 (15-CH_3), 34.6 (2-CH_2 or 3-CH_2), 29.9 (2-CH_2 or 3-CH_2), 20.9 (11-CH_2).

ν_{max} / cm^{-1} (compressed solid) 1669 (s), 1439 (m), 1179 (s), 1125 (s), 839 (s), 799 (s), 723 (s).

ESI-HRMS for $C_{34}H_{51}N_6^+$ $[MH]^+$ calcd: 543.4170, found 543.4169.

Melting point Decomp. > 180 °C.

5 NMR spectra of tested compounds

Compound 1: ^{1}H NMR (500 MHz, DMSO)

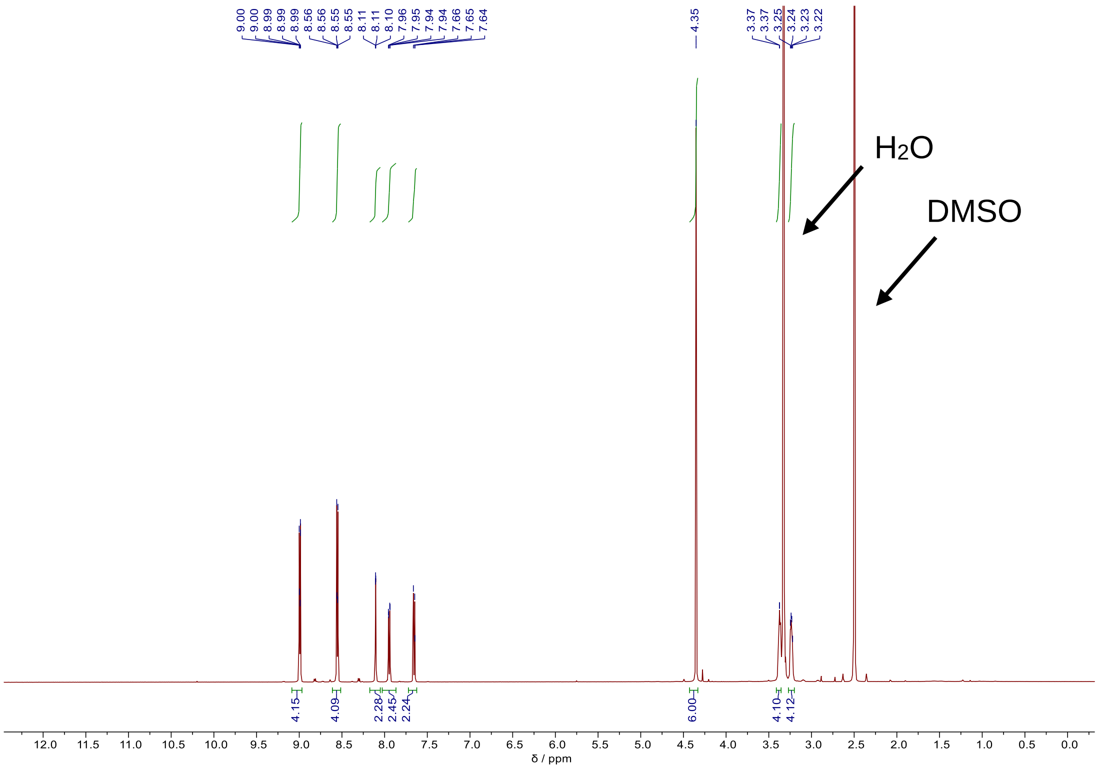

Compound 1: ^{13}C NMR (126 MHz, DMSO)

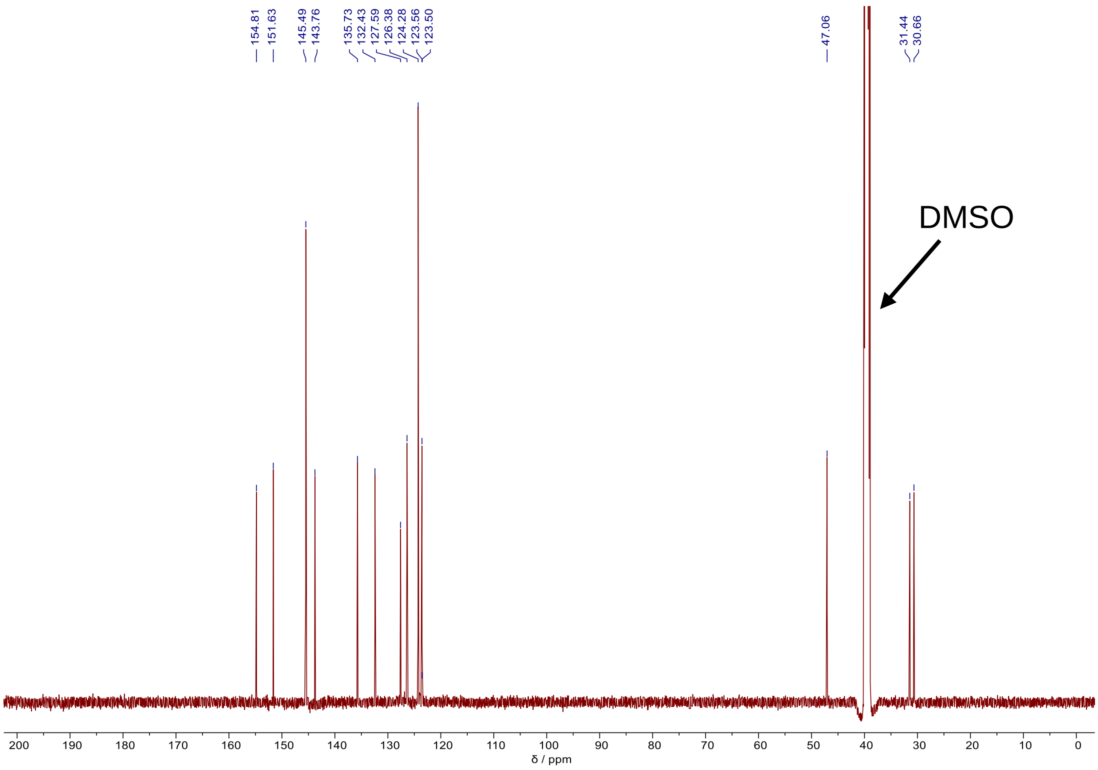

Compound 2: ^{1}H NMR (500 MHz, DMSO)

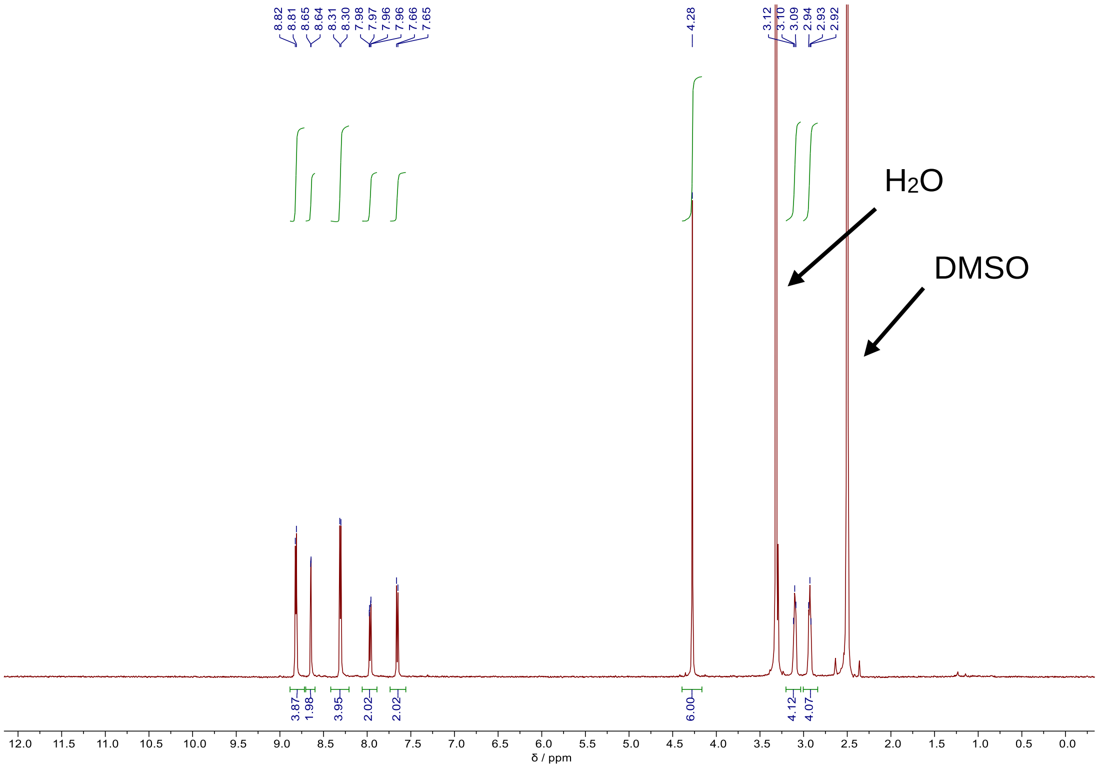

Compound 2: ^{13}C NMR (126 MHz, DMSO)

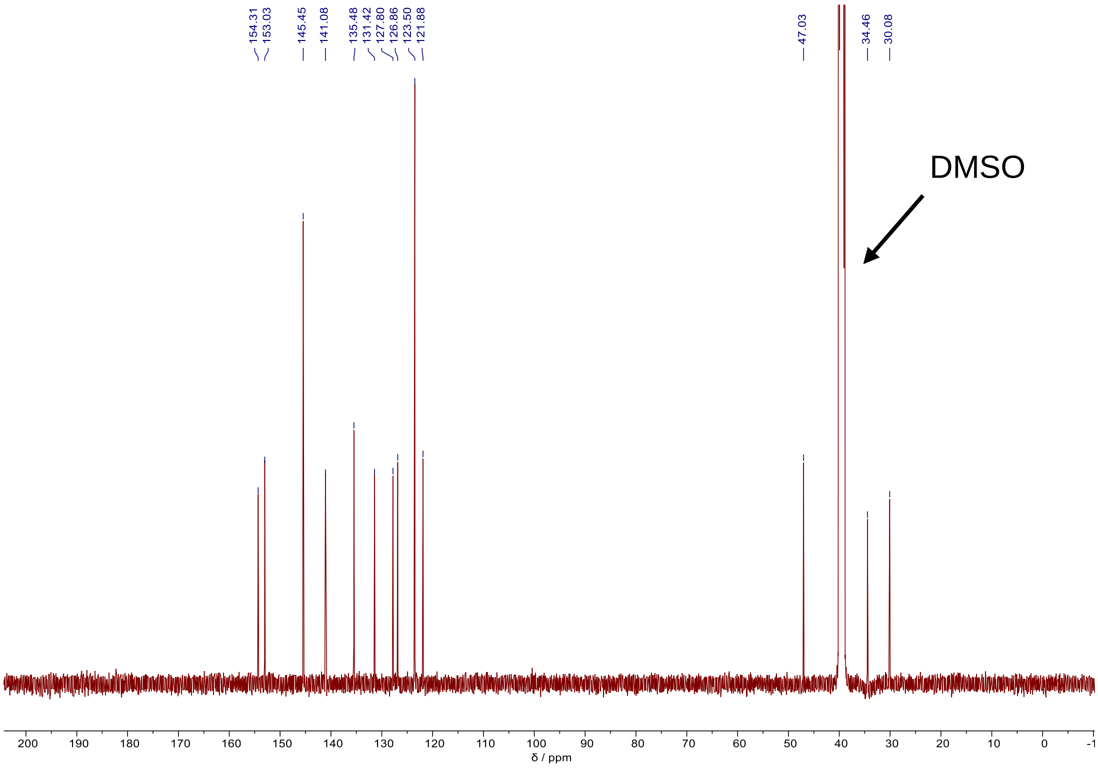

Compound 3: ^{1}H NMR (500 MHz, DMSO)

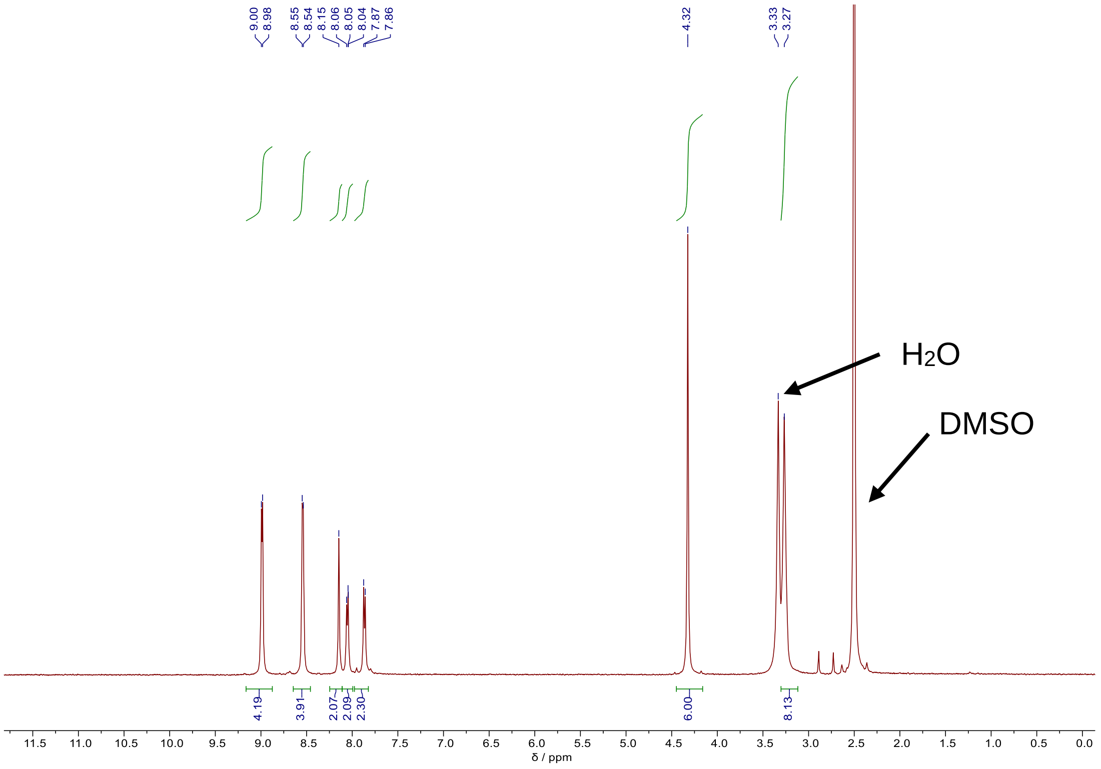

Compound 3: ^{13}C NMR (126 MHz, DMSO)

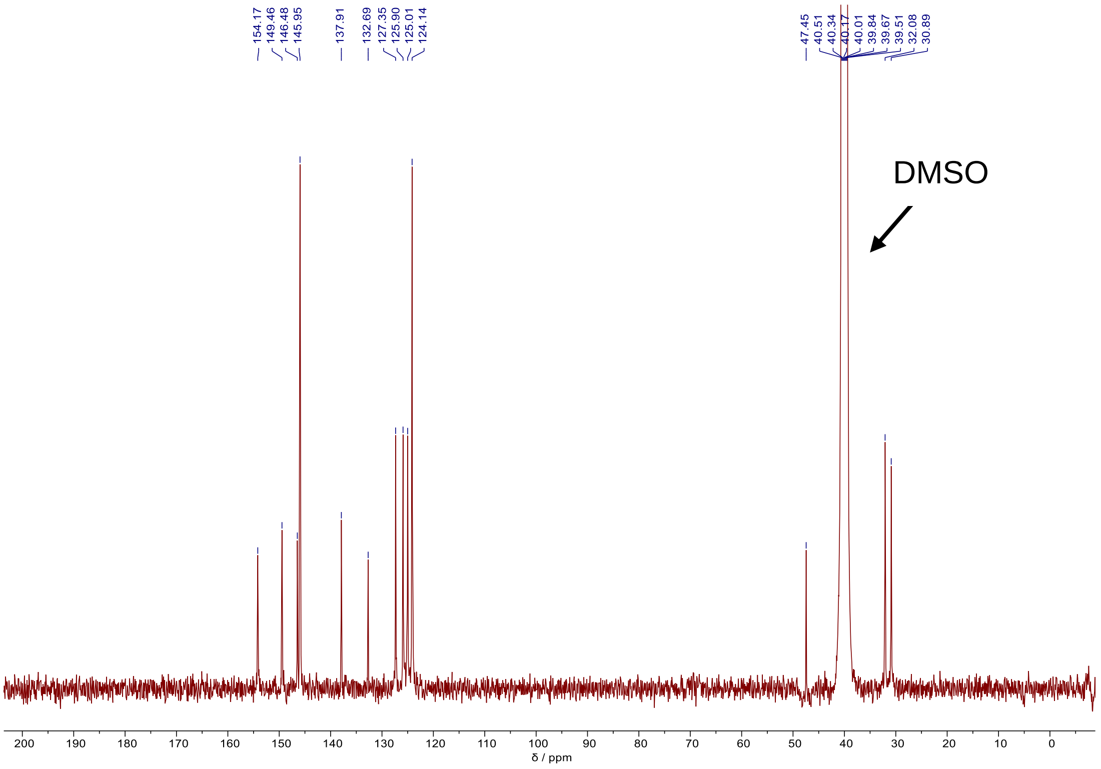

Compound 4: ^{1}H NMR (500 MHz, D_2O)

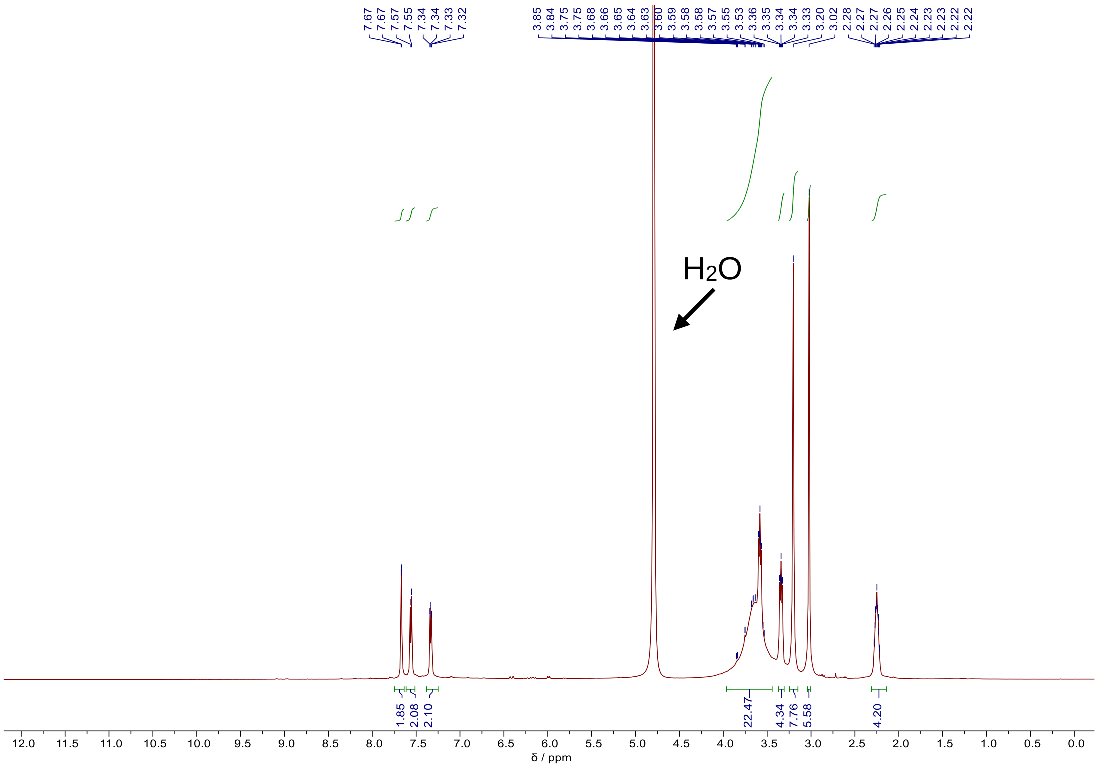

Compound 4: ^{13}C NMR (126 MHz, D_2O)

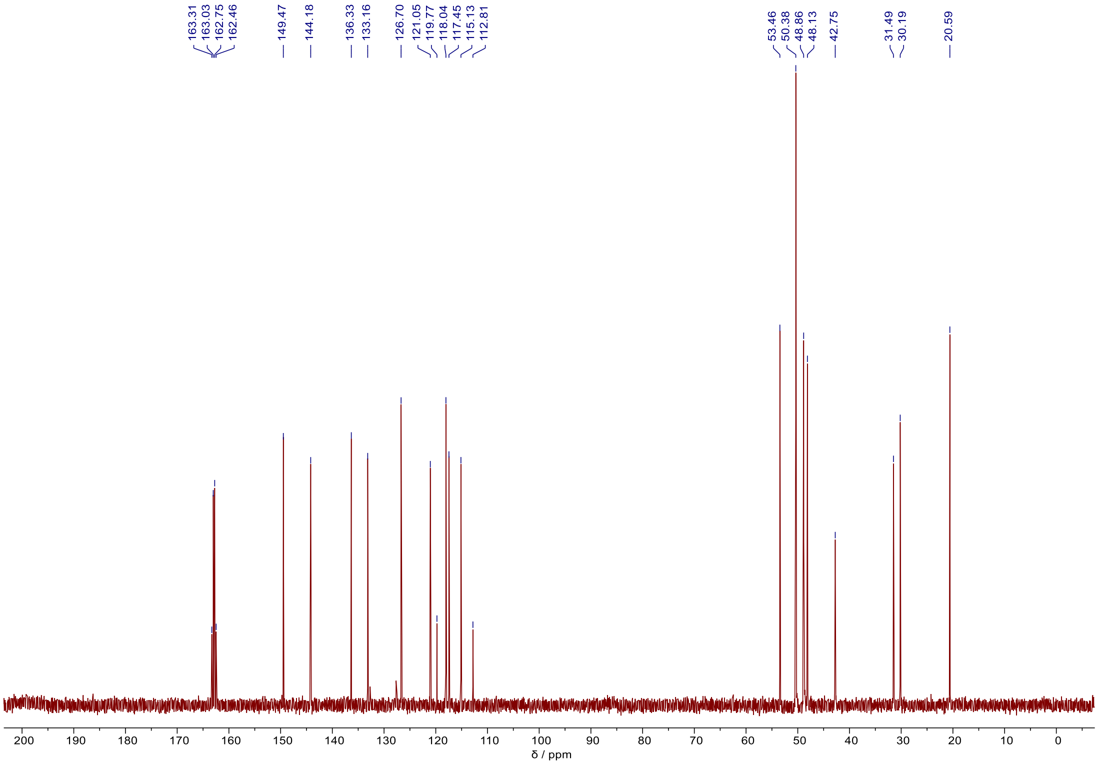

Compound 5: ^{1}H NMR (500 MHz, D_2O)

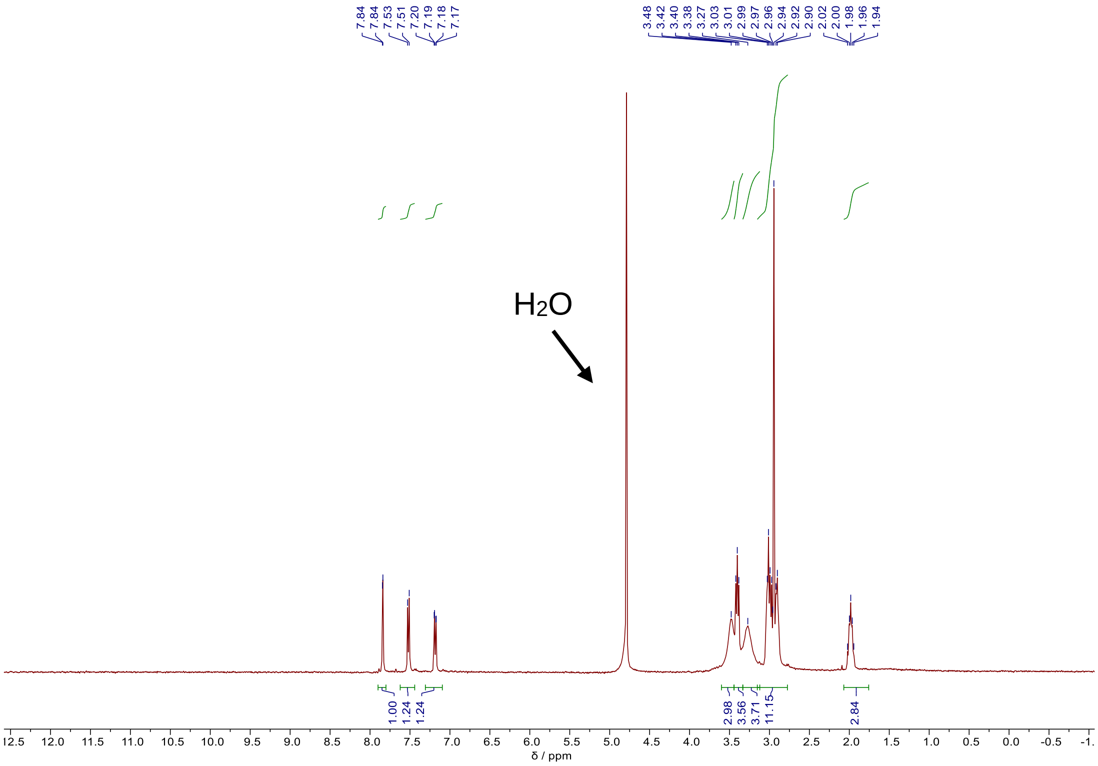

Compound 5: ^{13}C NMR (126 MHz, D_2O

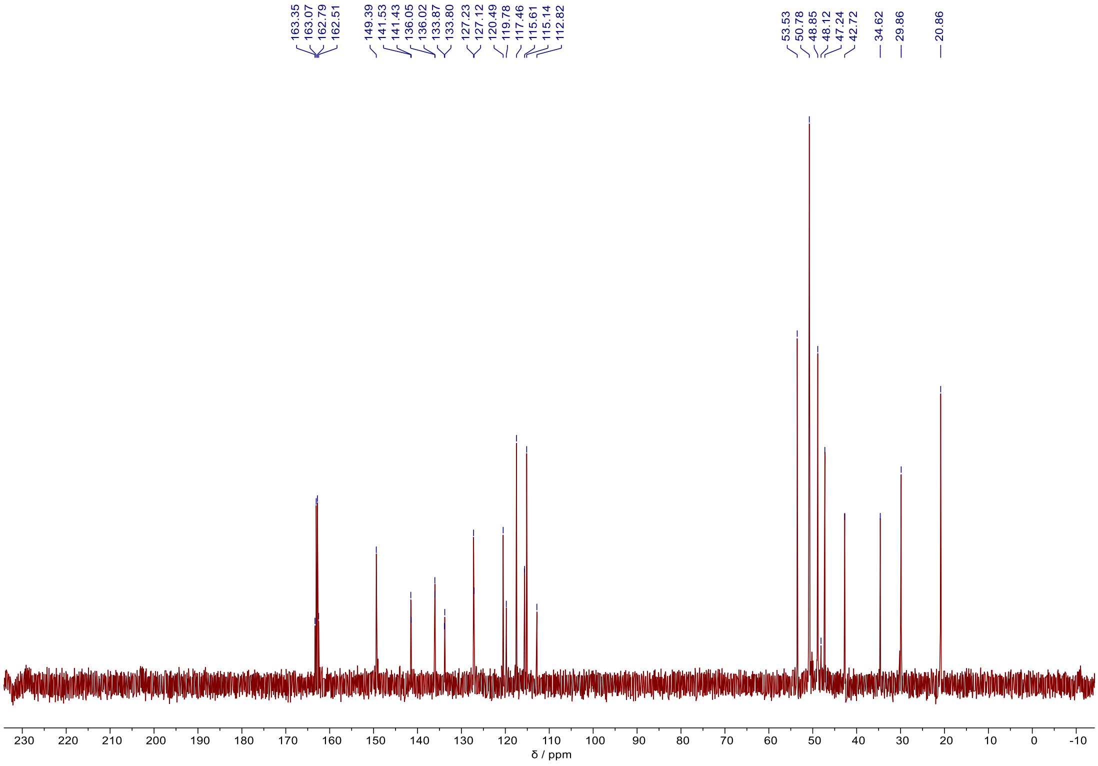

6 NMR spectra of novel synthetic intermediates

Compound *p*,(*E*)-6: ^{1}H NMR (500 MHz, D_2O)

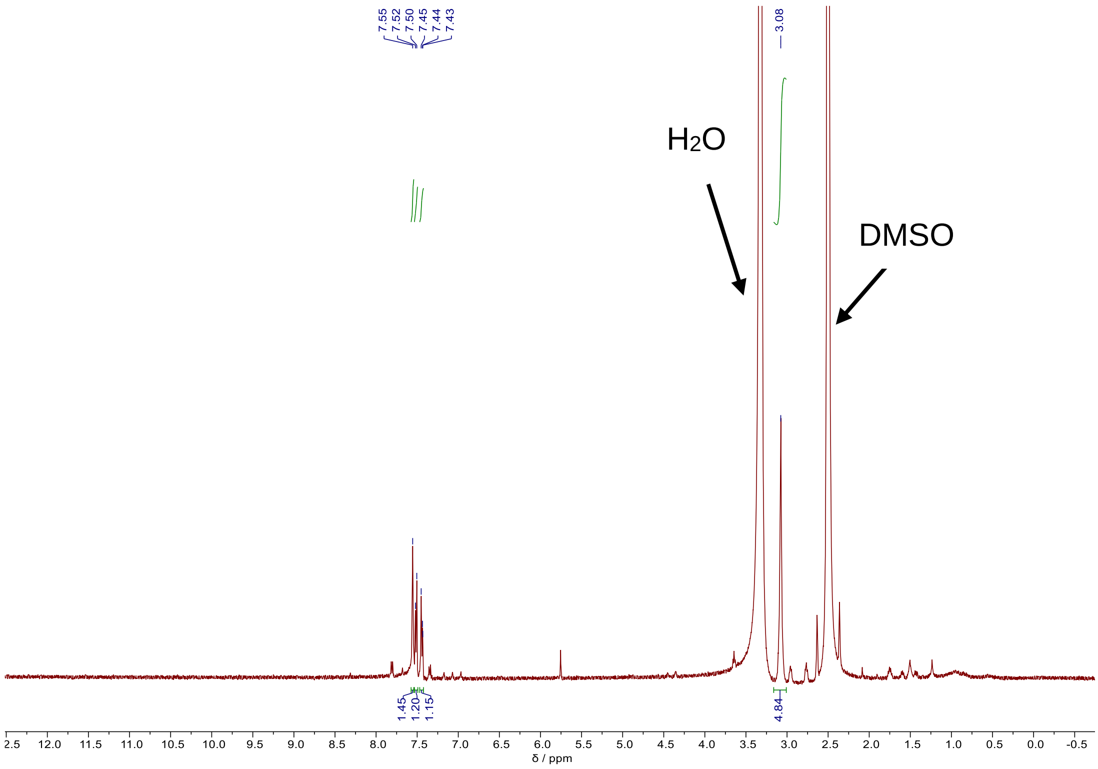

Compound *p*,(*E*)-6: ^{13}C NMR (126 MHz, D_2O)

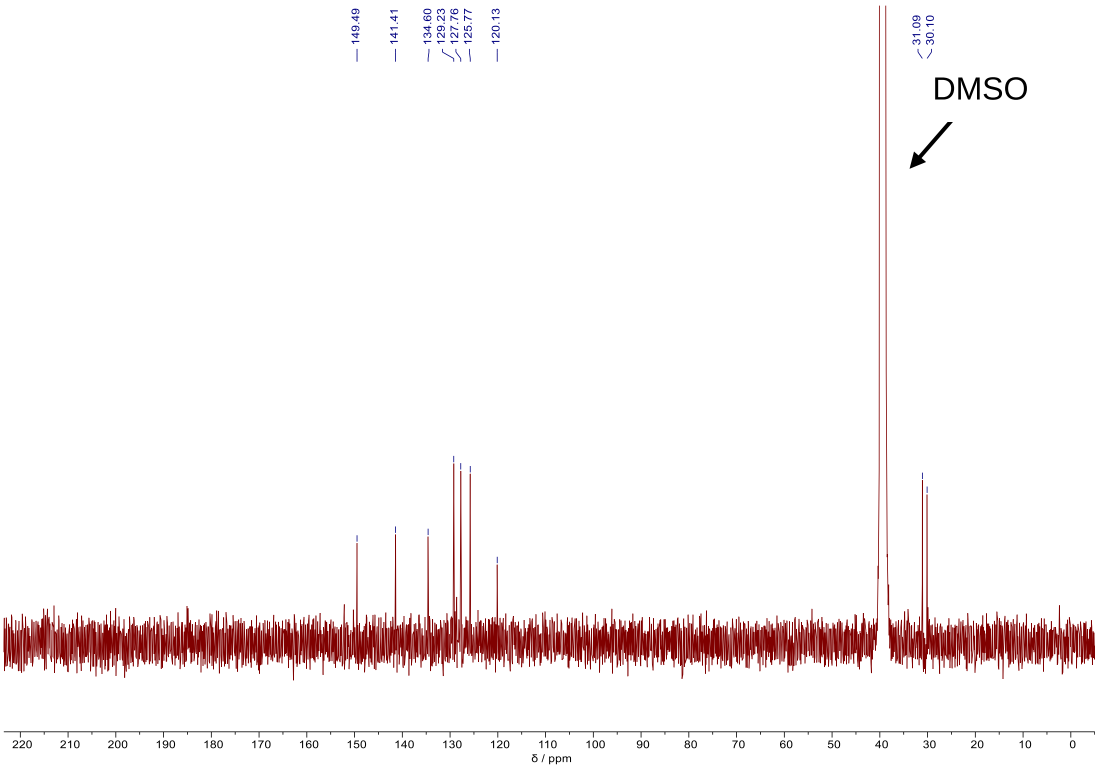

Compound *p,(E)*-9: ^{1}H NMR (500 MHz, $CDCl_3$)

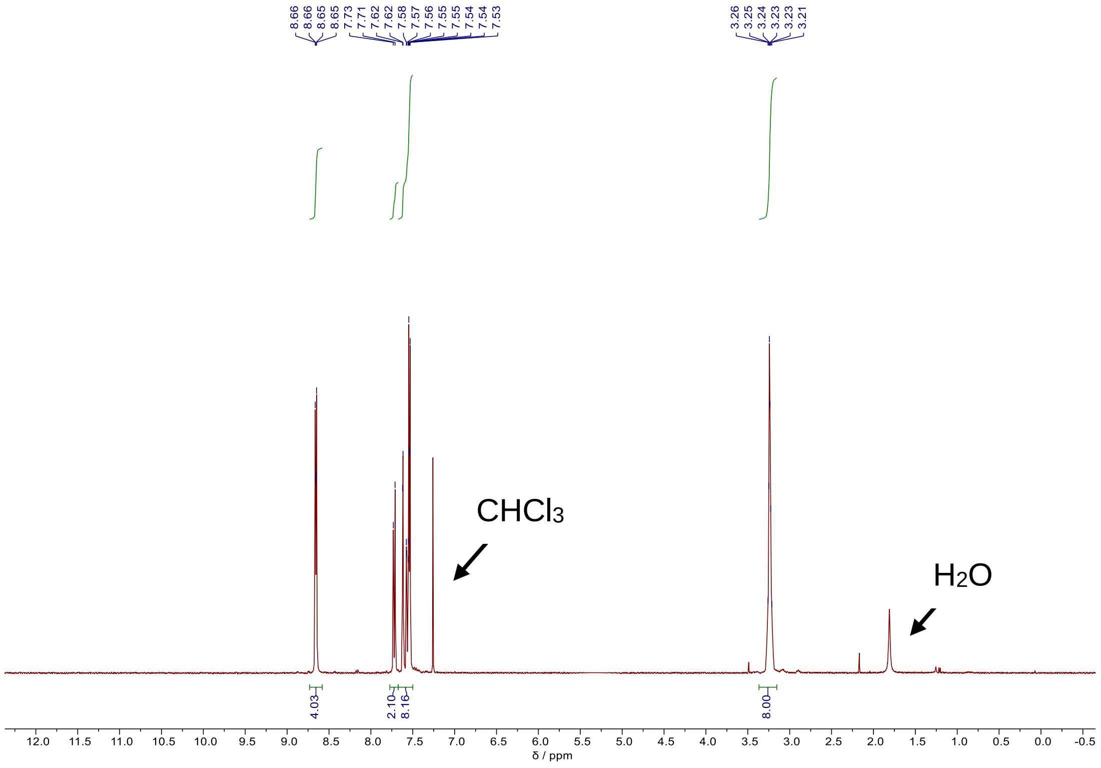

Compound *p,(E)*-9: ^{13}C NMR (126 MHz, $CDCl_3$)

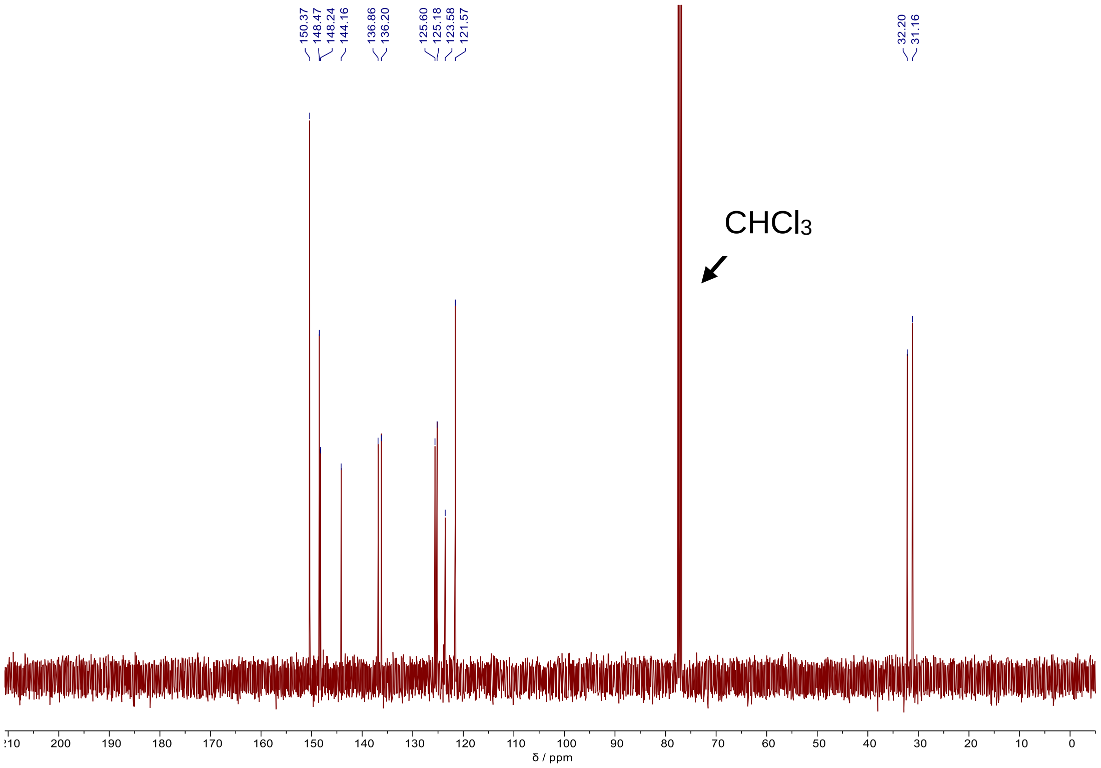

7 References

[1] A. De Cian, L. Guittat, M. Kaiser, B. Saccà, S. Amrane, A. Bourdoncle, P. Alberti, M. P. Teulade-Fichou, L. Lacroix, J. L. Mergny, *Methods* **2007**, *42*, 183–195.

[2] K. N. Luu, A. T. Phan, V. Kuryavyi, L. Lacroix, D. J. Patel, *J. Am. Chem. Soc.* **2006**, *128*, 9963–9970.

[3] P. Thordarson, *Chem. Soc. Rev.* **2011**, *40*, 1305–1323.

[4] L. Hahn, N. J. Buurma, L. H. Gade, *Chem. - A Eur. J.* **2016**, *22*, 6314–6322.

[5] E. Belmonte-Reche, M. Martínez-García, A. Guédin, M. Zuffo, M. Arévalo-Ruiz, F. Doria, J. Campos-Salinas, M. Maynadier, J. J. López-Rubio, M. Freccero, et al., *J. Med. Chem.* **2018**, *61*, 1231–1240.

[6] E. M. Larson, D. J. Doughman, D. S. Gregerson, W. F. Obritsch, *Investig. Ophthalmol. Vis. Sci.* **1997**, *38*, 1929–1933.

[7] J. M. Pérez-Victoria, B. I. Bavchvarov, I. R. Torrecillas, M. Martínez-García, C. López-Martín, M. Campillo, S. Castanys, F. Gamarro, *Antimicrob. Agents Chemother.* **2011**, *55*, 3838–3844.

[8] M. P. O'Hagan, S. Haldar, M. Duchi, T. A. A. Oliver, A. J. Mulholland, J. C. Morales, M. C. Galan, *Angew. Chemie Int. Ed.* **2019**, *58*, 4334–4338.

[9] A. B. Pangborn, M. A. Giardello, R. H. Grubbs, R. K. Rosen, F. J. Timmers, *Organometallics* **1996**, *15*, 1518–1520.

[10] W. C. Still, M. Kahn, A. Mitra, *J. Org. Chem.* **1978**, *43*, 2923–2925.

[11] S. J. Wezenberg, B. L. Feringa, *Org. Lett.* **2017**, *19*, 324–327.
